# Probing the Hydrogen-Bonding Environment of Individual Bases in DNA Duplexes with Isotope-Edited Infrared Spectroscopy

**DOI:** 10.1021/acs.jpcb.1c01351

**Published:** 2021-07-08

**Authors:** Robert J. Fick, Amy Y. Liu, Felix Nussbaumer, Christoph Kreutz, Atul Rangadurai, Yu Xu, Roger D. Sommer, Honglue Shi, Steve Scheiner, Allison L. Stelling

**Affiliations:** † Department of Chemistry and Biochemistry, The University of Texas at Dallas, Richardson, Texas 75080, United States; ‡ Department of Biochemistry, Duke University Medical Center, Durham, North Carolina 27710, United States; § Institute of Organic Chemistry and Center for Molecular Biosciences Innsbruck (CMBI), University of Innsbruck, Innsbruck 6020, Austria; ∥ Department of Chemistry, Duke University, Durham, North Carolina 27710, United States; ⊥ Molecular Education, Technology, and Research Innovation Center, North Carolina State University, Raleigh, North Carolina 27695, United States; # Department of Chemistry and Biochemistry, Utah State University, Logan, Utah 84322, United States

## Abstract

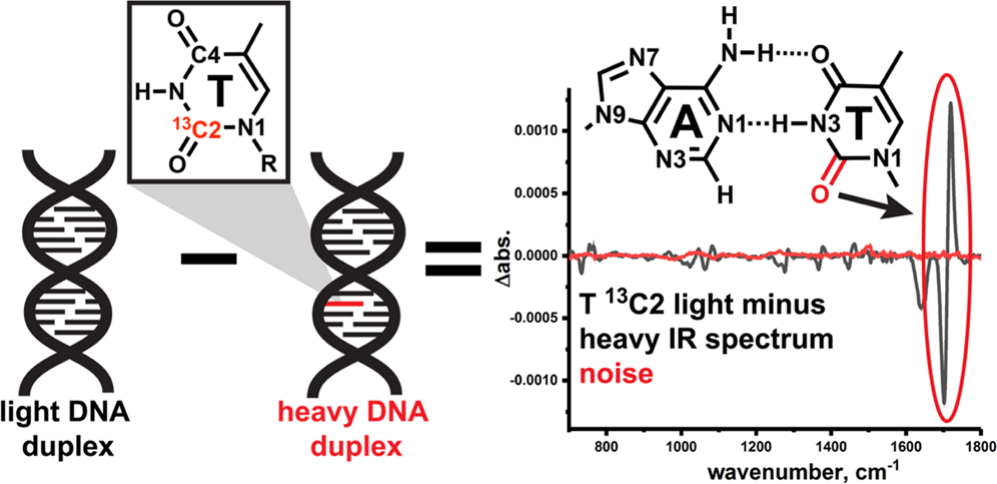

Measuring the strength of the hydrogen bonds between DNA base pairs is of vital importance for understanding how our genetic code is physically accessed and recognized in cells, particularly during replication and transcription. Therefore, it is important to develop probes for these key hydrogen bonds (H-bonds) that dictate events critical to cellular function, such as the localized melting of DNA. The vibrations of carbonyl bonds are well-known probes of their H-bonding environment, and their signals can be observed with infrared (IR) spectroscopy. Yet, pinpointing a single bond of interest in the complex IR spectrum of DNA is challenging due to the large number of carbonyl signals that overlap with each other. Here, we develop a method using isotope editing and infrared (IR) spectroscopy to isolate IR signals from the thymine (T) C2=O carbonyl. We use solvatochromatic studies to show that the TC2=O signal’s position in the IR spectrum is sensitive to the H-bonding capacity of the solvent. Our results indicate that C2=O of a single T base within DNA duplexes experiences weak H-bonding interactions. This finding is consistent with the existence of a third, noncanonical CH···O H-bond between adenine and thymine in both Watson–Crick and Hoogsteen base pairs in DNA.

## 1 Introduction

**Figure 1:**
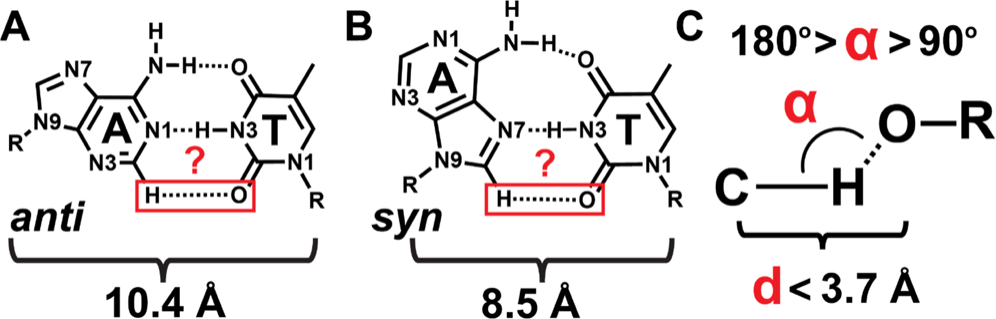
CH···O H-bonding in A–T DNA base pairs. (A) Potential, third CH···O H-bond in Watson–Crick and (B) Hoogsteen A–T DNA base pairs. (C) Typically used geometric definition for CH···O H-bonds.

Determining the hydrogen bonding (H-bonding) status of individual bonds in nucleic acids is key to understanding their structure, dynamics, and biological functions. This includes recognition by proteins, localized melting required for transcription and replication of DNA, and determining how specific sequences can be targeted with drugs. H-bonds are also critical for determining the complex tertiary structures formed by RNA. Understanding how key H-bonding interactions impact the three-dimensional structure of DNA is essential for determining how our chemical code is recognized by cellular machinery on a molecular level. This knowledge is critical for unraveling processes key to vitality, including replication and transcription, particularly, as it is the dysregulation of these processes that drives genetic diseases such as cancer. Yet, measuring the strength of individual hydrogen bonds (H-bonds) in DNA duplexes between the adenine (A)–thymine (T) and guanine–cytosine base pairs proposed by Watson and Crick^1^ (Figure 1A) in the 1950s remains a challenge, particularly in the native solution environment.

While the Protein Data Bank (PDB) contains thousands of DNA and DNA/protein structures at angstrom-level resolution, these structures can be profoundly impacted by the crystal packing. For example, there are a number of AT-rich DNA duplexes that crystallize as all-Hoogsteen base pairs^2,3^ rather than the canonical Watson–Crick pairs. In Hoogsteen base pairs^4,5^ (Figure 1B), the A or G base is flipped 180° from its anti configuration to syn, forming a completely new set of H-bonds with the T or C and exposing the Watson–Crick face of the purine base. However, many of these all-Hoogsteen duplexes are in fact Watson–Crick when examined in solution NMR,^6^ highlighting a need for methods sensitive to the molecular structure of individual DNA bases that can be performed in the native aqueous environment.

Two of the predominantly used molecularly resolved methods for detecting H-bonding in solution are NMR and vibrational spectroscopies. While NMR is exquisitely sensitive to the structure and electrostatic environment of individual bases, size limitations and chemical exchange render detection and measurement of H-bonds in large DNA/protein complexes challenging. Infrared (IR) spectroscopy is one of the few solution-state methods sensitive enough to changes in H-bonding and molecular structure that it can, for example, distinguish between Watson–Crick and Hoogsteen base pairs, even in large DNA/protein complexes.^7^ The frequencies of carbonyl stretching vibrations in particular are well-known^8−11^ to change when in strong vs weak H-bonds. C=O stretches can serve as sensitive probes for their immediate electrostatic environment^12−14^ and tend to have strong IR intensities that fall in a spectral window clear of signals due to the other molecular bonds. Carbonyl stretches have a long history of use for probing important H-bonding interactions in biological systems using vibrational spectroscopy, particularly as changes in C=O positions can be directly related to the enthalpy of H-bond formation,^10,15−18^ provided suitable calibrations are performed. For example, the C=O bond of *S*-acetyl-CoA was used as a probe to measure the degree of H-bonding to the acyl group when the cofactor was bound to α-chymotrypsin.^16^

DNA has been extensively studied by both one-^19−22^ and two-dimensional^23−28^ IR spectroscopy to probe the ground states and time-resolved methods^29−31^ to probe excited electronic states. However, three out of four of the bases contain at least one C=O bond, resulting in a highly overlapped region between 1600 and 1700 cm^–1^ in nucleic acid IR spectra. This renders detection of a particular C=O signal from any single base for use as a molecular probe of the base’s local electrostatic environment quite challenging. Here, we overcome this challenge using site-specifically incorporated, isotopically labeled bases and spectral subtraction to dramatically reduce spectral overlap. Isotope labeling is a well-established method for assigning signals to vibrations from particular atomic groups in congested IR and Raman spectra of polyatomic molecules. A multitude of studies have incorporated ^13^C=O or ^13^C=^18^O labels into the amide backbone of proteins^32,33^ and peptides,^34−38^ or of small-molecule enzymatic substrates,^15,39,40^ to analyze the hydrogen bonding status of these groups. A recent study used fully ^15^N- and ^13^C-labeled DNA bases to examine coupling in G-quadruplexes with ultrafast 2DIR spectroscopy.^41^

**Figure 2:**
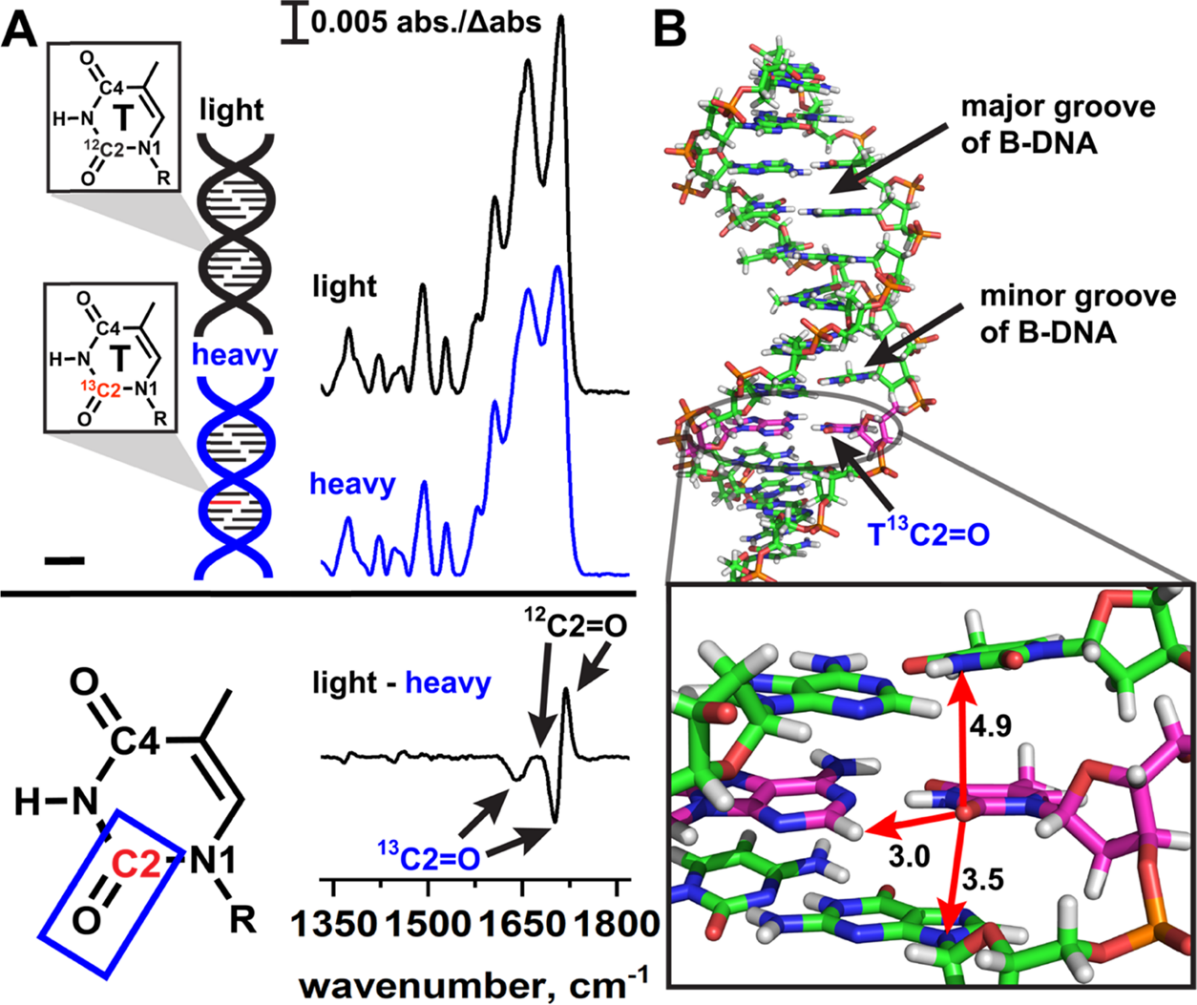
Isotope editing and spectral subtraction isolate isotope signals from individual T bases in DNA duplexes. (A) In isotope editing experiments, an isotopically labeled base is site-specifically incorporated into a DNA duplex (heavy, blue) and the heavy IR spectrum is subtracted from the IR spectrum of a light duplex (black), which has an identical sequence. In the “light–heavy” subtraction spectrum, signals due to the light ^12^C=O appear as positive bands, and signals due to the heavy ^13^C=O appear as negative bands. Signals that arise from vibrations in which the C2 atom does not participate are canceled out, thus reducing spectral congestion so that C=O stretching of a specific bond of an individual DNA base can be observed in the duplex environment. (B) B-form DNA duplex illustrating the local environment of a TC2=O bond. Arrows indicate nearby functional groups on neighboring bases that may impact the stretching frequency. Distances are in angstrom.

A specific application that exploits isotope labeling is isotope editing experiments in which the IR (or Raman) spectrum of the heavy isotope is subtracted from its light counterpart (see the schematic in Figure 2A), canceling out overlapping signals from modes due to other bonds in the molecule. It is more straightforward to ascribe the resulting difference signals to specific functional groups in large molecules, particularly those vibrations which are well-localized to two or three atoms. In nucleic acids, previous studies examined the conformation of adenine bases within DNA duplexes^42^ and DNA/protein complexes^43^ using deuterium-labeled adenine bases. More recent studies employed isotope editing experiments using commercially available, fully ^15^N- and ^13^C-labeled RNA bases to track incorporation of a single labeled base using Raman microscopy during an RNA polymerase reaction.^44−46^ However, to the best of our knowledge, to date, no isotope editing studies have been performed that label an individual C=O in nucleic acids.

Here, we develop an IR-based isotope editing method to report the first detection of the TC2=O frequency in individual DNA bases when in duplexes containing two different types of A–T base pairs: Watson–Crick^1^ and Hoogsteen.^5,47^ For these initial experiments, we focused on the C2 atom of T as the C2=O stretch as, based on earlier^48^ and more recent^28,49^ experimental studies, we expected it to be localized to the C and O atoms, making it a suitable reporter for its immediate chemical environment. The TC2=O bond is located in the minor groove of B-form DNA (Figure 2B) and is therefore expected to be sensitive to interactions with adenine C2–H, any solvent waters or ions that may bind to the minor groove, and stacking interactions from the adjacent bases immediately above or below it.

Here, we present the “light minus heavy” isotope subtraction spectra (Figure 2A) for a series of DNA duplexes, each 12 base pairs in length. These specific sequences were chosen for two main reasons. First, they have been extensively examined with solution NMR methods and, second, two out of the three sequences can form A–T Hoogsteen base pairs when either bound to a drug (echinomycin) or when chemically modified to enforce Hoogsteen pairing, thus allowing us to determine if the stretching is sensitive to changes in the sequence context, in base pairing, and in the minor groove environment. Our third sequence serves as a control for the drug-bound A–T Hoogsteen base pairs as this sequence binds to the drug but maintains Watson–Crick A–T base pairing when bound. Thus, this initial series of duplexes allows us to test the sensitivity of the C2=O bond to two different types of base pairing, Watson–Crick and Hoogsteen, in systems that have been validated to form each under solution conditions by NMR spectroscopy.

We compare the position of the TC2=O stretching frequency in these DNA duplexes to that from the thymine base in solvents with different H-bonding capacities. Collectively, our results indicate that TC2=O in DNA duplexes experiences a weak H-bonding interaction. This may be due to the presence of a weak CH···O H-bond (Figure 1C) in A–T Watson–Crick and Hoogsteen DNA base pairs, which is suggested to exist in computational studies^50,51^ but has been vigorously disputed.^52−54^ Alternatively, the interaction may arise due to a weakly bound water molecule in the minor groove of the DNA duplexes, which has been observed in crystal structures,^55−63^ solution NMR^64,65^ and 2DIR^66−69^ studies, and molecular dynamics caluclations.^70,71^

Our results are a powerful proof of concept of how isotope editing with labeled DNA bases is a robust, solution-state detection method capable of pinpointing individual chemical bonds encased in very large macromolecular complexes. As carbonyls are present in every nucleic acid save for adenine, we anticipate that this method can be applied to study the electrostatic environment of individual bases in a broad range of DNA- and RNA-containing systems, including their complexes with drugs and proteins.

## 2 Materials and Methods

### 2.1 Chemicals

Unless otherwise indicated, all chemicals for buffers, thymine base small-molecule mimics, echinomycin, and solvents were purchased from Sigma-Aldrich. Natural-abundance DNA duplexes were purchased from Integrated DNA Technologies. Fully labeled TA-DNA was purchased from Yale-Keck.

### 2.2 Synthesis of ^13^C2 -Labeled Thymine Phosphoramidite

**Scheme 1:**
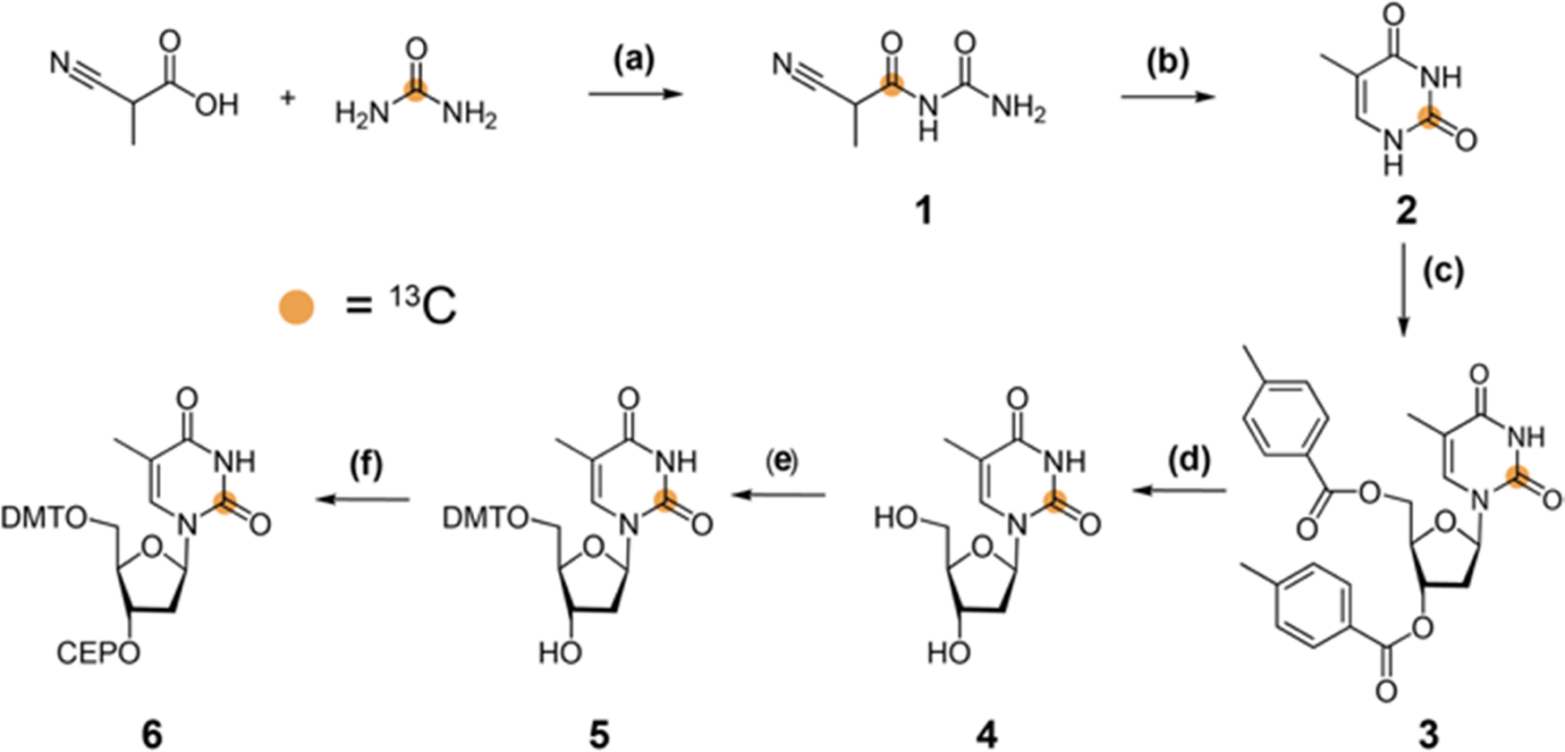
Synthesis of 2-^13^C-Labeled Thymidine Phosphoramidite (a) Acetic anhydride, 90 °C, 1 h, and 81%; (b) Pd/BaSO_4_ 5%, H_2_, in AcOH/H_2_O, 16 h, rt, and 67%; (c) **1**. HMDS, TMS-Cl, 120 °C, 16 h, then Hoffer′s α-chlorosugar, in CHCl_3_, 4 h, 40 °C, and 56%; (d) CH_3_NH_2_ in EtOH 8 M, 16 h, RT, and 88%; (e) DMT-Cl, DMAP, in pyridine, 3 h, rt, and 71%; and (f) CEP-Cl, DIPEA, in CH_2_Cl_2_, RT, 3 h, and 74%. DMT, 4,4′-dimethoxytrityl and CEP, 3′-*O*-(*N*,*N*-diisopropyl phosphoramidite).

Scheme 1 shows the chemical structure of the synthetic intermediates and an overview of the route. See the Supporting Information Methods section for experimental details, yields, and spectroscopic characterization for compounds **1**–**6**.

### 2.3 IR Experiments

#### 2.3.1 General

All DNA IR measurements were performed at DNA concentrations between 5 and 10 mM in a sodium phosphate buffer (25 mM sodium phosphate, 15 mM NaCl) at pH 6.9. IR measurements were performed with a PerkinElmer Frontier FTIR spectrometer using an NB MCT detector, a PIKE MIRacle diamond ATR attachment, and a CaF_2_ coverslip as previously described.^7,72^ In general, 128 scans at 4 cm^–1^ resolution were taken for each measurement and the average of minimum three measurements was used for averaging in OriginPro. All DNA duplex spectra were baselined with the Spectrum multipoint baselining function and normalized to the phosphate band at ∼1080 cm^–1^. Isotope editing subtractions were performed in the Spectrum (PerkinElmer) software using the subtraction function and the average of at least three independent replicates was exported and used for averaging in OriginPro.

#### 2.3.2 Oligonucleotide Synthesis

Single strands containing the T ^13^C2 isotope were synthesized on a MerMade synthesizer using standard protocols.^73^ The ^13^C thymine C2-labeled single strands were synthesized in-house using a MerMade 6 Oligo synthesizer. ^13^C C2-labeled thymine phosphoramidites, standard DNA phosphoramidites (*n*-ibu-G, *n*-bz-A, *n*-ac-C, and T Chemgenes), and columns (1000 Å from Bioautomation) were used with a coupling time of 1 min, with the final 5′-dimethoxytrityl (DMT) group retained during synthesis. The oligonucleotides were cleaved from the supports (1 μmol) using ∼1 mL of AMA (1:1 ratio of ammonium hydroxide and methylamine) for 30 min and deprotected at room temperature for 2 h. The single strands were then purified using Glen-Pak DNA cartridges, with ethanol precipitated and resuspended in water for subsequent annealing.

All unlabeled DNA duplexes were annealed as previously described.^72^ Briefly, complementary single-stranded DNA were combined in 0.5 mL of ddH_2_O or buffer, allowed to anneal at 95 °C for ∼5 min, and cooled at ambient temperature for at least 45 min. The samples were then concentrated and exchanged at least three times into the sodium phosphate buffer using 0.5 mL Amicon (Millipore) with a MW cutoff of 10 kDa. All IR measurements used the flowthrough from the previous exchange as the background spectrum.

#### 2.3.3 Echinomycin Binding

Echinomycin reactions with both TA- and AT-DNA duplexes were performed as previously described^74−80^ with minor modifications. Echinomycin was dissolved in methanol (∼5 mL methanol per 1 echinomycin) and the concentration was determined via UV–vis spectroscopy using the extinction coefficient at 325 nm (11.5 mM^–1^ cm^–1^).^81^ A three-fold molar excess of echinomycin was used for binding. To ensure binding, DNA was diluted such that it was at an equal volume with the methanol/echinomycin solution and shaken at room temperature for at least 2 h. The mixture was then divided into 1.5 mL Eppendorf tubes and allowed to slowly air-dry overnight. Once dry, the reaction was resuspended using ddH_2_O in a volume equal to that of the buffer and concentrated in 0.5 mL Amicon to a volume of ∼30 to 50 μL. This was then exchanged into the sodium phosphate buffer at least three times before measurement with IR. The extent of binding was determined by inspection of the symmetric phosphate stretching band at 1085 cm^–1^ in the IR spectra of complexes, which shows a characteristic loss of the shoulders at ∼1020 and 1050 cm^–1^ and gain at ∼1040 cm^–1^ upon binding. Additional markers for drug binding in the IR include an increase in the intensity in the C=O stretching region at ∼1650 cm^–1^ and the red shift to the antisymmetric phosphate stretching by ∼6 cm^–1^.

#### 2.3.4 Solvatochromatic IR Experiments with Thymine Bases

For experiments in water, both dTTP (∼100 mM) and a single-stranded poly-T 12-mer (∼3 mM) were used to determine the C2=O positions. For dimethylformamide and *d*_6_-DMSO measurements, 1-methylthymine was used to increase solubility. Briefly, 2–3 mg of 1-methylthymine was dissolved by heating at ∼95 °C for less than 10 min, and 16 scans were used to avoid sample evaporation. The highest frequency C=O signal, which usually lies between 1650 and 1800 cm^–1^, was then plotted against the solvent acceptor number and a linear regression was performed in Excel.

### 2.4 Crystallization of 9-Methyladenine and 1-Methylthymine

Crystals of the dimer were grown by combining equal amounts (∼1 mL) of 0.1 M solutions of 9-methyladenine and 1-methylthymine in a 5 dram vial. The volume was reduced by slow evaporation to approximately one-half. The vial was then covered and the cap loosened to allow for further evaporation to 1/4 volume. This process yielded large rod-shaped crystals. Typical crystals had a height and a width of 0.1 and 0.2 mm, respectively, and varied in length from 0.5 to 1.2 mm. A representative crystal (0.096 × 0.212 × 0.572 mm^3^) was selected and mounted on a 0.1 mm MiTeGen mount.

Diffraction data were collected using a Bruker-Nonius X8 Kappa APEX II diffractometer and Mo Kα radiation (λ = 0.71073 Å) from a fine-focus sealed tube and graphite monochromator. Corrections were applied using SADABS (Bruker-AXS Inc. (2019)), Madison Wisconsin. The structure was solved using direct methods and refined using least-squares refinement of *F*^2^ and the SHELXTL software package (Bruker-AXS Inc. (2019), Madison Wisconsin). All nonhydrogen atoms were refined anisotropically. Most H atoms were visible in the difference map in the later stages of refinement but were placed at standard calculated positions. Alkyl and aryl H atoms were allowed to ride on the parent atom position with isotropic displacement parameters set to 1.2 and 1.5 times that of the parent atom, respectively. Positions for hydrogen atoms involved in close contacts of interest for this study were allowed to be refined while maintaining the isotropic displacement parameter restraint.

The dimer crystallizes in space group *P*2_1_/*m*. General information is tabulated in Table S1. The molecule sits on the mirror plane. Close contacts between A and T are consistent with those of earlier structures^47,82−84^ with negligible variation in intermolecular contacts. For this work, the rotational disorder of the methyl groups was modeled across the mirror plane. Structure determination confirms that the crystals used for this work are indeed consistent with the structure used to analyze the spectroscopic and electronic properties in this study.

### 2.5 Ab Initio Calculations

Ab initio calculations made use of the MP2 treatment of electron correlation along with the polarized 6-31+G* basis set, within the context of the Gaussian-09 set of codes.^85^ Heavy atoms were frozen in their experimentally determined positions, while H atom positions were fully optimized. Interaction energies were evaluated as the difference in energy between each base pair and the energy sum of the individual monomers and then corrected for the basis set superposition error by the standard counterpoise protocol.^86^ The densities of the H-bond critical points were evaluated by the AIMALL program.^87^

## 3 Results

### 3.1 Isotope Editing Pinpoints TC2=O Signals from Individual Bases in Large DNA Duplexes and DNA/Drug Complexe

#### 3.1.1 Detection of Watson–Crick TC2=O Signals in Two DNA Duplexes with Isotope Editing

For performing isotope editing experiments, there is a choice between two different T isotopes. The commercially available, fully ^13^C- and ^15^N-labeled nucleic acid bases used in previous IR and Raman studies are labeled at every carbon and nitrogen atom, including the sugar and backbone carbons. Isotopically substituting many atoms results in a heavier mass change, which may result in a stronger difference spectrum and thus provide higher contrast in large DNA/protein complexes. However, the difference signal obtained from multiple isotope incorporation may be more complex than that of a duplex containing only one or two labeled atoms due to the multiple mass changes. This mass effect might make ascribing physical interpretations to specific atomic groups, such as an individual C=O bond, very challenging as it would be difficult to determine the extent to which the observed signal is due to a vibration that is localized to the carbonyl of interest. We therefore first sought to compare the difference spectra taken between light and heavy DNA duplexes when using the fully labeled T and a T with only one ^13^C label inserted at the C2 position, which was chosen as the C2=O stretching vibration is more likely to be localized to the C and O atoms^88^ and therefore be a faithful reporter of the H-bonding environment of the minor groove.

**Figure 3:**
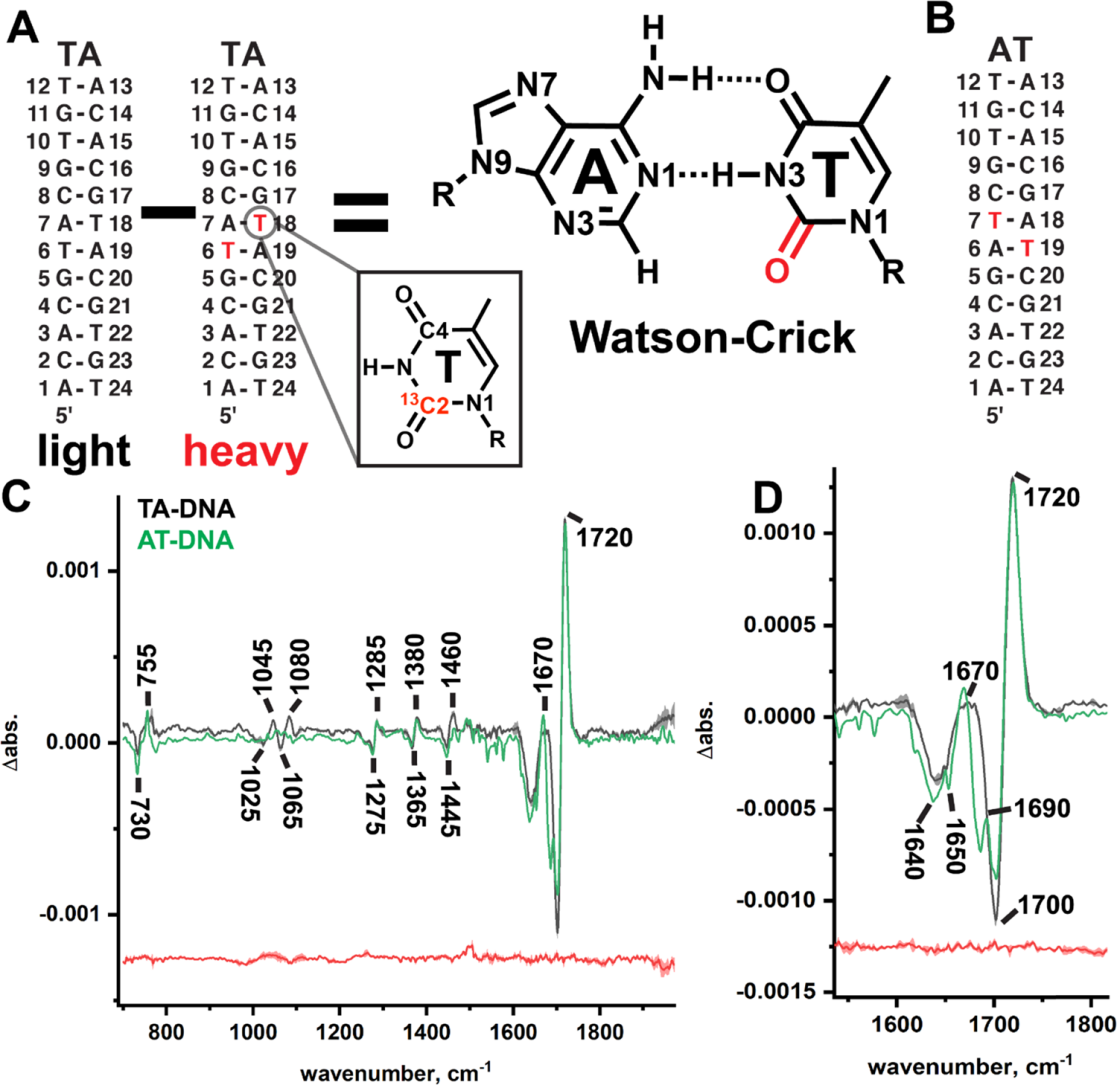
Isotope editing experiments pinpoint TC2=O IR signals in Watson–Crick base pairs in DNA duplexes. The sequence of the palindromes TA-DNA (A) and AT-DNA (B). Labeled T bases are indicated in red. (C) Light minus heavy IR difference spectrum of ^13^C2-labeled TA-DNA (black) and AT-DNA (green). Positive bands from ^12^C2=O and negative bands from ^13^C2=O are labeled. (D) Enhanced view of the carbonyl region in (C) showing splitting in the minor signal of the AT-DNA duplex. A representative noise line (replicate minus replicate for TA-DNA) is shown in red. The mean of four independent measurements is shown in bold, and the standard deviation in shade.

We therefore site-specifically installed fully ^13^C- and ^15^N-labeled and single-atom ^13^C2-labeled thymine bases into a TA-DNA duplex (Figure S1). As this sequence is a palindrome (Figure 3A), each duplex now contained two labeled T bases. The spectrum of the unlabeled (light) TA-DNA duplex was subtracted from that of the labeled (heavy) one (Figure S1A) to produce a difference spectrum for each type of label (Figure S1B) in which positive signals are due to the light isotope vibrations, and negative signals from the heavy ones. The difference signal in the fully labeled T is more pronounced but significantly more complex than that of the single-atom ^13^C- and ^15^N-labeled T due to the multiple mass changes, rendering any potential physical interpretation of the data challenging. To circumvent this complexity, we decided to develop singly ^13^C2-labeled T as a probe for the H-bonding environment of A–T base pairs in DNA duplexes using two well-studied^74^ palindromes, TA-DNA (Figure 3A) and AT-DNA (Figure 3B). The major positive signal in both ^13^C2-labeled TA-DNA and AT-DNA (Figures 3C,D and S1) is an intense band at ∼1720 cm^–1^ that we preliminarily assign to the localized stretching of TC2=O in a B-form duplex based on values in the experimental literature for isotopically labeled T bases^28,48,49,89^ and theoretical calculations.^88^ The negative signal at ∼1700 cm^–1^ is downshifted by the expected amount (∼20 cm^–1^) for a ^12^C to ^13^C mass shift,^37,38^ which is well in excess of our instrument’s resolution (set at 4 cm^–1^).

We also observe a minor positive signal in each duplex centered at ∼1670 cm^–1^, whose intensity is roughly one quarter that of the major signal in both duplexes. Interestingly, the two different sequences show differences in this minor signal: in TA-DNA, the signals are broad and in AT-DNA, the signals appear to be splitting into two separate components (Figure 3D). We preliminarily assign the minor signal to delocalized C4=O/C5=C6 stretching with amplitude from the labeled ^13^C2 atom based on experimental isotope shifts^48^ for the isolated T base and theoretical calculations of the A–T base pair.^88^ This is consistent with 2DIR studies^24,28^ of AT-rich Watson–Crick duplexes that observe four bands in this region due to stretching from both bases, three of which are thought to be due to vibrations localized to the T base and one to adenine. In deuterated buffer, the three from the T base are located at 1690 (C2=O stretching, T_2R_, which we observe in water as our “major signal” at 1720 cm^–1^), 1660 (C4=O stretching, T_4R_), 1640 (T ring stretching, T_R_), and 1630 cm^–1^ (adenine ring stretching, A_R1_). It may be noted that we observe T C5=C6 stretching with our T ^13^C2 label, resulting in three signals contributing to our difference spectrum. The C=C stretching mode in the T mononucleotide^49^ (T_R_) was found to be fairly localized to the C5 and C6 atoms, but its character may change upon entry into the duplex environment, allowing for it to be observed with our ^13^C2 isotope. Thus, our “minor signal” in both duplexes may be a composite band containing two overlapping signals: one from T_R_ and the other from T_4S_. This assignment, and the dependence of the splitting of this signal on the sequence context, will be investigated more deeply in future studies using T ^13^C4-labeled bases.

The T_R_ stretching mode is also thought to be more sensitive to interstrand base pairing than to intrastrand stacking interactions with its flanking base pairs, as this mode shifts down to 1630 cm^–1^ upon heating past the duplex melting temperature.^28^ Yet, as mentioned, our isotope-edited spectra for these two fully base-paired duplexes show a few differences, the most prominent of which can be seen in the minor positive signal centered at 1670 cm^–1^. In TA-DNA, this signal is quite broad with a band width at half maximum of ∼30 cm^–1^. In AT-DNA, the minor ∼1670 cm^–1^ signal is split into two components: a dominant one at ∼1665 cm^–1^ and a weak shoulder at ∼1690 cm^–1^ (Figure 3D).

There are a few physical models that might account for the splitting observed in the minor signal. One is that the minor signal splitting in the T base in AT-DNA experiences two separate H-bonding environments at its C2=O, giving rise to two populations of strongly and weakly bound carbonyls with two different band positions. A previous study using NMR chemical shifts and N–H coupling constants^90^ has indicated that the hydrogen bond strength of A–T base pairs may be sequence-dependent and thus our AT-DNA sequence may have weak base pair H-bonding, which in turn can result in the breaking of the Watson–Crick H-bond between C4=O and the adenine −NH_2_. This results in a small signal due to a population of C4=O that is blue-shifted owing to formation of a nonbonded C4=O. Another possibility that may account for the splitting might arise from the two different sequence contexts experienced by TC4=O in each duplex due to the different sequence contexts for the T base. In TA-DNA, both C2-labeled T bases are adjacent to guanines and are in proximity (about 4 Å) to the guanine C6=O. In AT-DNA, the T bases are flanked with cytosines and are at a much greater distance from the cytosine’s C4=O (about 6 Å). If the T_R_ mode is sensitive to stacking and base pairing, then, different sequence contexts would be expected to shift this mode down, revealing the T_4S_ mode at 1690 cm^–1^ in AT-DNA.

A third potential model is that the sequence context impacts the character of base modes in DNA duplexes, and thus changes in through-bond coupling between T base modes cannot be rejected completely as the potential source of the splitting. Meanwhile, through-bond coupling due to the frequency shift upon mass labeling is unlikely to be the origin for the splitting, as if this was the case, then the splitting ought to be independent of the sequence context. Future studies using a range of sequence contexts and isotope labels, abasic sites, temperature variance, and 2DIR will aid in discriminating between these physical models.

The edited spectra of TA-DNA and AT-DNA overlay quite well below ∼1600 cm^–1^, although one additional difference is observed: TA-DNA has two weak signals at 1080 and 1045 cm^–1^ that are not present in the spectrum of AT-DNA. Signals at this frequency have been attributed to delocalized T ring motions,^48,89^ and it is possible that the sequence context has altered the mode’s character such that the C2 atom now contributes a higher percentage of amplitude to the mode in TA-DNA.

#### 3.1.2 Detection of TC2=O IR Signals in Drug-Bound Hoogsteen and Watson–Crick A–T Base Pairs

**Figure 4:**
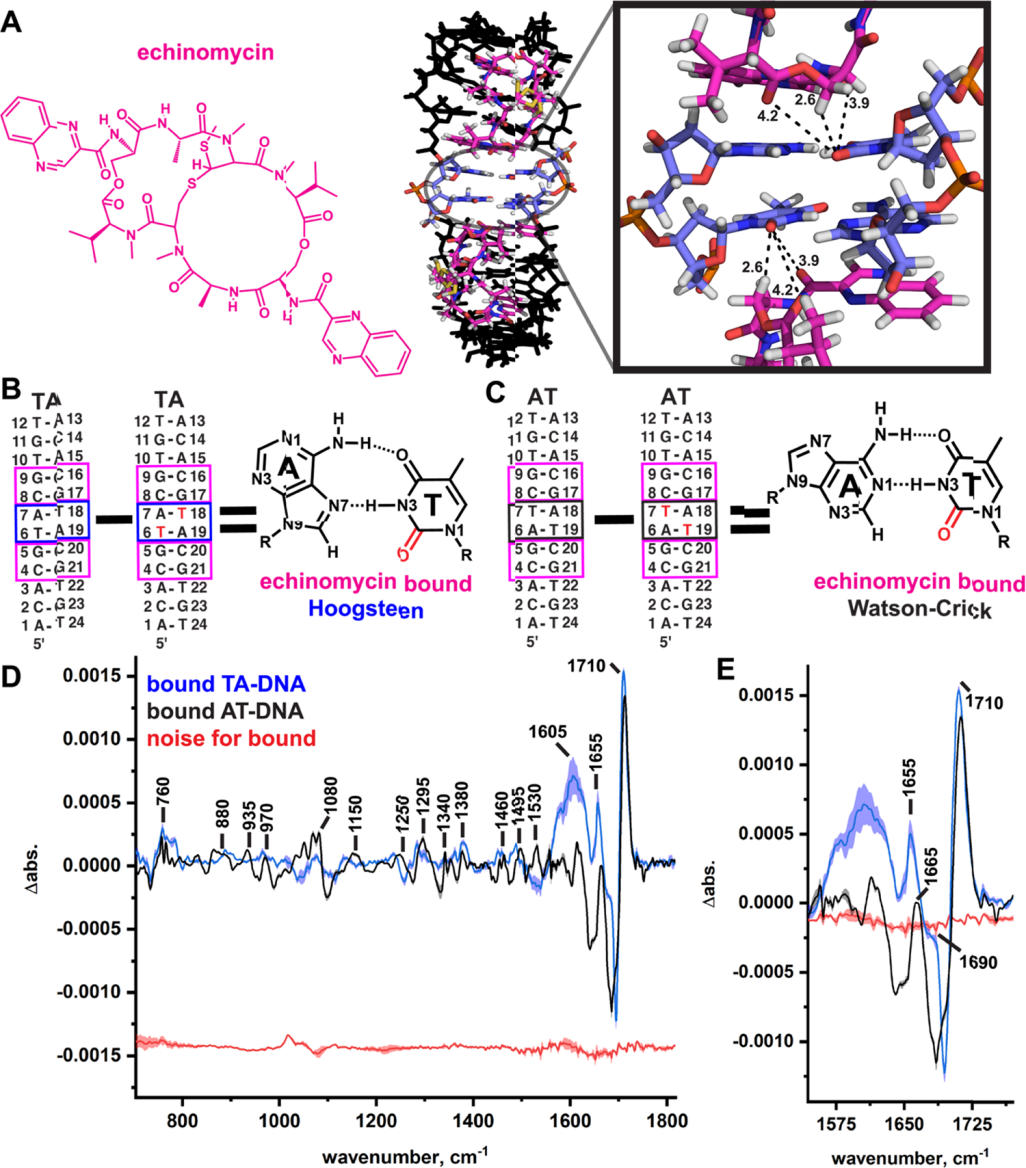
Isolation of the TC2=O stretching signal in a DNA/drug complex with Hoogsteen base pairs. (A) Chemical structure of echinomycin (magenta) and a crystal structure of the bound TA-DNA (PDBID: 1XVN),^91^ which induces the formation of two Hoogsteen base pairs (blue). Dashes indicate functional groups from echinomycin close to the labeled carbonyls (distances in angstrom). (B) and (C) GC-step binding sites of echinomycin in TA-DNA are boxed (magenta). Echinomycin was bound to both the light and heavy TA-DNA duplexes and their difference spectra calculated to isolate signals from the two A–T Hoogsteen (T6/T18, blue box) base pairs’ TC2=O stretching signals (see the chemical structure; TC2=O is in red). (D) and (E) Light minus heavy IR difference spectrum of the ^13^C2-labeled DNA/echinomycin complex with the positive signals from the light isotope labeled. Representative noise lines (replicate minus replicate) are shown in red. The mean of three to four independent measurements is shown in bold, and the standard deviation in shade.

To test if isotope editing at the T C2 position can isolate signals from atoms in a large DNA/drug complex, and to determine if Hoogsteen base pair formation of A–T base pairs impacts the TC2=O signal, we examined two DNA duplexes when bound to the drug echinomycin (Figure 4A),^74,78,79,91^ a peptide inhibitor^92^ that possesses anticancer, antiviral, and antibacterial activities. In the TA-DNA duplex, two echinomycin molecules bind to two GC sites (Figure 4A) and induce the formation of tandem A–T Hoogsteen base pairs (Figure 4B) that are sandwiched between the drug-binding sites. In contrast, when echinomycin is bound to the AT-DNA duplex, these tandem AT base pairs maintain Watson–Crick pairing^76,77,79^ (Figure 4C). Thus, these two duplexes allow us to test the sensitivity of our edited IR signal to two different types of DNA base pairs, Watson–Crick and Hoogsteen, while simultaneously controlling the presence of the drug as it binds each sequence in an identical manner. Additionally, as echinomycin is an intercalator, binding ought to also remove contributions from stacking: the tandem A–T base pairs are effectively isolated, yet still in a DNA duplex, and this allows us to determine the impact of stacking on the splitting observed in the free duplexes by comparison of the AT-DNA free and drug-bound spectra.

Labeled and unlabeled TA-DNA and AT-DNA duplexes were bound to echinomycin and the light spectrum subtracted from the T ^13^C2-labeled spectrum. The extent of binding was determined by examining changes in the IR signals from the DNA backbone and C=O bonds from both echinomycin and DNA (Figure S2) that are characteristic of echinomycin binding,^72^ specifically, a decrease in the shoulder at ∼1050 cm^–1^, an increase at ∼1040 cm^–1^, an increase at 1740 cm^–1^, and large increases at ∼1700 cm^–1^ and between 1650 and 1610 cm^–1^. Our results (Figures 3C and S3A) show that the difference signal is strong in both the edited spectra, even in the large DNA/drug complex, confirming that our method can be used to isolate C=O signals from specific bases in large, macromolecular complexes.

The major, most intense positive signal observed in the isotope-edited spectra of both drug-bound duplexes are red-shifted by ∼10 cm^–1^ from their unbound signals from 1720 cm^–1^ down to ∼1710 cm^–1^ (Figures 4D,E and S3A,B). This red shift may be due to duplex unwinding upon drug intercalation, allowing water or buffer salts to more deeply penetrate the minor groove, or due to interactions with the drug itself. As can be seen from the crystal structure, there are two carbonyls from the drug relatively close (∼4 Å) to each TC2=O in TA-DNA (Figure 4A), as well as a −CH_2_ group (∼2.6 Å). The bond dipoles of the drug’s C=O groups are orientated opposite to those of the TC2=Os. Thus, if they interacted with each other, then a blue shift and not a red shift would be expected.^93^ We therefore conclude that the source of the ∼10 cm^–1^ red shift in both drug-bound duplexes relative to the free DNA likely arises from increased H-bonding from solvent waters to C2=O as the minor groove is widened by echinomycin. This red shift may also arise from destacking due to drug intercalation, which effectively “deletes” interactions between the T base and the neighboring base; however, shifts due to stacking are not anticipated to result in such a strong red shift.^94^ These hypotheses will be tested further using D_2_O exchange in future studies to determine if there is an observable isotope shift and inserting abasic sites flanking the isotopically labeled base, which ought to remove any stacking.

Interestingly, the minor signal observed at ∼1670 cm^–1^ in the free TA-DNA duplex dramatically splits when echinomycin is bound and Hoogsteen base pairs are formed into two components at ∼1690 and ∼1655 cm^–1^ (see Figure S3A for an overlay of the drug-bound and free TA-DNA). Meanwhile the AT-DNA/echinomycin complex, which is bound to the drug in an identical fashion to TA-DNA but preserves the Watson–Crick base pairs when bound, exhibits a single minor signal at ∼1665 cm^–1^ (see Figure S3B for an overlay of the drug-bound and free AT-DNA). As with the free duplexes, the physical origins of this minor-band splitting in the drug-induced Hoogsteen base pairs may arise from different mechanisms (assuming that this mode arises from the T_4S_ and T_R_ modes, with minor participation from the ^13^C2-labeled atom). If we ascribe the ∼1690 cm^–1^ signal once again to T_4S_, this would imply that the T_R_ mode downshifts by ∼15 cm^–1^ in the drug-induced Hoogsteen base pair formed in the TA-DNA/drug complex. This is in line with a previous study,^28^ indicating that this T ring mode is sensitive to base pairing.

Many 2DIR studies^23,25,26^ have illustrated that coupling between vibrational modes is a major determinant of why IR spectroscopy is sensitive to DNA structure and base paring. Splitting in duplexes may arise from both interactions with modes located within flanking bases and interactions with modes located on its base-pairing partner.^25,94^ A recent theoretical study^94^ indicates that in WC A–T pairs, T modes are coupled to ring C=C bond vibrations in the partnering adenine, and this has also been observed using 2DIR spectroscopy.^24,28,95−97^ This coupling would likely be impacted by the flipping of the adenine base from anti to syn in the Hoogsteen base pair, which switches the orientation of the adenine ring. Future experiments on these duplexes using ^13^C4-labeled T will aid not only in the interpretation of this splitting but also in the validation of its suitability for use as a marker band for A–T Hoogsteen base pairs.

Finally, it is worth noting another signal centered at ∼1605 cm^–1^ (broad in TA-DNA and narrow in AT-DNA) that is present in both bound duplexes and absent in the free duplexes, the origins of which are not clear. This may be due to adenine −NH_2_ scissoring, as computational studies indicate that base modes in general can become highly delocalized upon base pairing^88^ and modes can contain contributions from both bases. Thus, our labeled T ^13^C2 atom may participate in adenine vibrations (such as amino scissoring) that would be expected to be impacted by drug binding. Future studies with ^13^C4=O isotope and isotopes on the adenine and T ring atoms and complimentary 2DIR studies will aid in the assignment of these minor signals and provide physical interpretations for the observed splitting.

#### 3.1.3 Detection of TC2=O Signals in Singly^13^C-Labeled Watson–Crick and m^1^A-Hoogsteen Base Pairs

**Figure 5:**
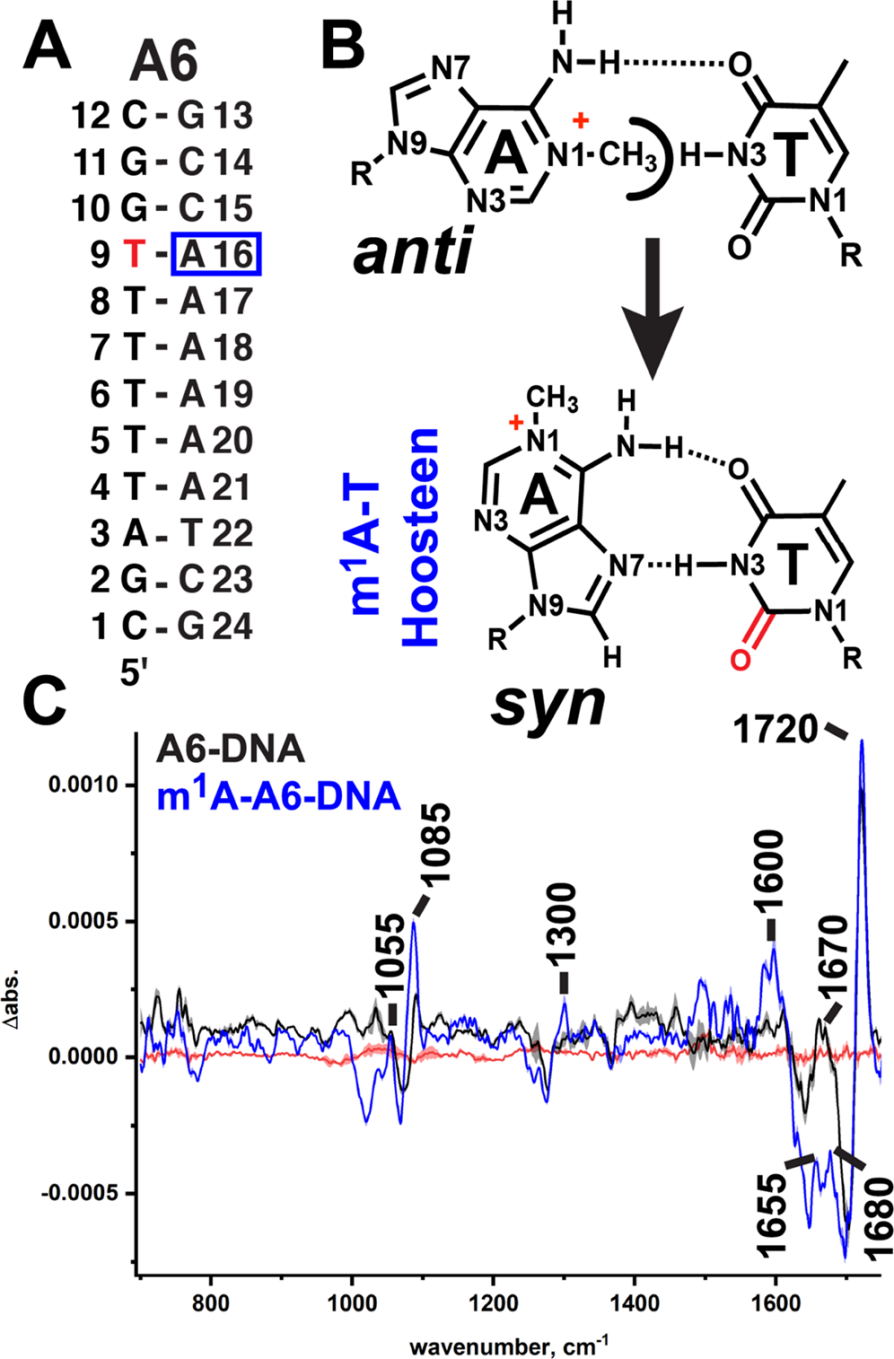
Isotope editing pinpoints a single T atom in two DNA duplexes. (A) Sequence of A6-DNA with the labeled T (T9) in red. (B) Chemical structure of N1-methyladenine (m^1^A) incorporated at A16, opposite the ^13^C2-labeled T (red). Methyl group on the adenine N1 has a steric clash with the T N3–H, resulting in the purine flipping over into its Hoogsteen form. (C) Light minus heavy difference spectra of A6-DNA (black) and m^1^A-A6-DNA (blue).

As mentioned, both the TA- and AT-DNA duplexes are palindromic and so contain two ^13^C-labeled carbon atoms. To test if the IR difference signal arising from a single ^13^C-labeled atom in a large 12-mer DNA duplex is detectable above the noise for the measurement and to examine if the observed frequency is altered when changing the sequence context, we incorporated a ^13^C2 T base into the A6-DNA duplex (Figure 5A). To determine if the splitting observed in the drug-induced Hoogsteens in TA-DNA arises from the drug or from Hoogsteen formation, we installed an N1-methyladenine (m^1^A, Figure 5B), which is a known form of DNA damage,^98^ opposite the labeled T base. The addition of the methyl on the N1 of adenine (Figure 5B) results in a positive charge being placed on the base, and a steric clash with the T N3–H forces it to flip 180° and form Hoogsteen H-bonds.^99−106^

In the isotope-edited spectra of T ^13^C2-labeled A6-DNA, the signal is ∼50% that of the doubly labeled T ^13^C2s in TA and AT-DNA but quite detectable above the noise for the measurement (Figure 5C). The A6-DNA difference spectrum is almost identical to that of TA-DNA (see Figure S4A for overlays of the A6-DNA, TA-DNA, and AT-DNA spectra), with a major signal at 1720 cm^–1^ and a broad minor signal at ∼1670 cm^–1^. In m^1^A-A6-DNA (Figure 5C), the major signal maintains its position at 1720 cm^–1^. As in the drug-bound, Hoogsteen-forming TA-DNA duplex, the minor signal at 1670 cm^–1^ is split into two bands (see Figure S4B for an overlay of m^1^A-A6-DNA with the bound TA-DNA), with one now at ∼1680 cm^–1^ and the other at ∼1655 cm^–1^. The ∼1680 cm^–1^ band is ∼10 cm^–1^ red-shifted from the signal observed in the nonchemically enforced Hoogsteen base pairs formed in the TA-DNA/echinomycin complex. This may be due to the positive charge on the chemically modified m^1^A base, which could increase the strength of the H-bond between TC4=O and the adenine −NH_2_ and in turn lower the frequency of the T_4S_ stretch.

Taking together the results from the m^1^A-A6-DNA duplex and from the free TA-DNA and AT-DNA duplexes, we conclude that Hoogsteen H-bonding does not alter the frequency of the major TC2=O stretching signal, whose position is thus far independent of the sequence context and base pairing but does appear to be sensitive to the H-bonding environment in the minor groove. Meanwhile, the strong red shift down to ∼1655 cm^–1^ observed in the minor signal in both drug-induced and chemically enforced A–T Hoogsteen base pairs (Figure S4B) is not observed in either drug-bound or free WC base pairs (see Figure S4A for overlays) and may arise solely from either a different coupling from T base modes to adenine ring vibrations in a syn vs an anti adenine or due to increased H-bonding at TC4=O in a chemical-enforced Hoogsteen base pair. Either way, this splitting may therefore be suitable for use as a marker band for detecting protein-bound A–T Hoogsteen pairs in solution. We note that our short duplexes have about equal AT-to-GC content (six A–T pairs in TA- and AT-DNA and seven A–T pairs in A6-DNA), and so studies that dramatically change the AT-to-GC ratios of the sequence along with studies on much longer duplexes must be performed to determine the limits of detection in our isotope-edited spectra.

### 3.2 Sensitivity of TC2=O IR Signals to the Solvent Environment

#### 3.2.1 Solvatochromatic Studies of TC2=O and Its Sensitivity to Solvent H-Bonding

The isotope editing experiments provide a means to measure the frequency of TC2=O in solution-state DNA duplexes, but calibration is required to determine if this band position changes as a function of the strength of H-bonding to C2=O. To provide an initial interpretation for the relative strength of interactions at TC2=O in a duplex environment, we measured IR spectra of the T base in solvents with different H-bonding abilities. We also crystallized the 9-methyladenine/1-methylthymine dimer from Hoogsteen’s original paper^5,47^ and measured its IR spectrum. (The IR spectrum of Hoogsteen A–T crystals was examined in earlier studies;^107,108^ however, the sample was either too thick for a transmission measurement in the 1800–1300 cm^–1^ region^109^ or only the amide stretching region was examined.)^108^ If this CH···O H-bond exists in Watson–Crick and/or Hoogsteen base pairs, then the frequency of C2=O ought to be greater than 1720 cm^–1^ in solvents with decreased H-bonding power, as measured by Gutmann’s H-bond acceptor number (AN)^110^ for the given solvent. Our solvatochromatic results are in line with those of previous studies using Raman spectroscopy,^111^ reporting that both T C=O stretching signals are sensitive to solvent H-bond strength.

**Figure 6:**
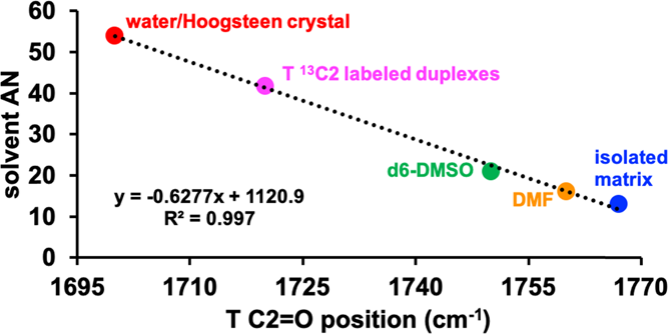
Acceptor numbers for A–T base pairs in DNA duplexes. A plot of the solvent’s Gutmann H-bond acceptor number (AN) vs the observed TC2=O stretching. Values for ^13^C2-labeled duplexes, the Hoogsteen crystal, and the isolated matrix N1-methylthymine were calculated based on the regression line derived from the observed values for the T base in water, dimethylformamide (DMF), and *d*_6_-dimethyl sulfoxide (*d*_6_-DMSO). The isolated matrix value is from N1-methylthymine in the study by Person and co-workers.^112^

The highest frequency signal of the T base (when not in a duplex) has been shown via isotope labeling studies to arise from localized C2=O stretching in its Raman and IR spectra.^48,89^ We plotted the highest positions (Figure S5A,B) vs the H-bond acceptor number of the solvent in which T was dissolved (Figure 6). We used the equation obtained from a linear regression of these data to calculate the AN values for the band positions that we observed via T ^13^C2 isotope editing in the DNA duplexes, the 9-methyladenine/1-methylthymine Hoogsteen crystal, and the isolated N1-methylthymine’s observed IR position in an argon matrix (from ref^112^^112^). We find that in solvents with diminished H-bonding power such as *d*_6_-DMSO (AN = 21), the C2=O signal lies at ∼1750 cm^–1^, while in strong H-bonding solvents like water (AN = 54), the signal is shifted down to ∼1695 cm^–1^.

In the 9-methyladenine/1-methylthymine dimer crystal, which crystallizes with Hoogsteen H-bonding (Figure S6A,B),^5,47^ the IR position of TC2=O is about the same as that observed in water (Figure S6C), which might indicate a very strong CH···O H-bond. However, in our structure, the measured distance between the carbon and oxygen atoms is 3.664 Å, which is just under the typical cutoff distance. Upon inspection of the crystal packing (Figure S6B), we observed another H-bonding interaction between TC2=O and an adenine −NH_2_ group from an adjacent unit cell, which is likely the source of the strong red shift in the Hoogsteen crystal and supports our hypothesis that TC2=O stretching is a robust probe of the H-bonding environment.

In summary, the frequency of C2=O is shifted down in energy by ∼30 cm^–1^ when in a Watson–Crick or a Hoogsteen H-bond in a DNA duplex from its position in *d*_6_-DMSO, indicating that C2=O experiences a H-bond interaction in DNA duplexes in both WC and Hoogsteen base pairs whose strength is less than that of water. This red shift in the paired duplex from the *d*_6_-DMSO and the isolation matrix values is consistent with either a CH···O interaction with the adenine C2/C8–H (Figure 6), which has been observed in nucleic acid crystal structures,^113^ or a weakly bound water molecule forming the spine of hydration^55−57,97^ in the minor groove of the duplexes.

### 3.3 Calculations of the CH···O Bond Strength for A–T Base Pairs

#### 3.3.1 Ab Initio Calculations of the CH···O Bond in A–T Base Pairs

To investigate the energetics of the CH···O interaction, we performed MP2 correlated calculations using several different A–T base pair coordinates. One set used the PDB coordinates (PDBID: 2ADW) for a DNA sequence bound to echinomycin that contained two duplexes in the unit cell, one of which had WC A–T pairs and the other had Hoogsteen pairs (Figure S7A), so that energies could be effectively compared when in drug-bound DNA. The other set used NMR coordinates^101,114^ for the A16–T9 base pair in the A6-DNA (PDBID: 5UZF) and m^1^A-A6-DNA (PDBID: 5UZI, methyl group has been removed) (Figure S7B) duplexes. Interaction energies were assessed as the difference between the energy of each base pair and the sum of the two monomers.

We evaluated the interaction energy of the Watson–Crick and Hoogsteen dimers (Table 1), which were then compared to two controls to estimate the strength of the CH···O bonds. In one control, the C2/8H···O interaction is abrogated by substitution of the C2 or C8 with a N atom (Figure S7C). This loss of the CH···O H-bond reduces the interaction energy by 2.2–3.8 kcal/mol. In the other control, we rotated the T base by 90° out of the joint plane, around the CH···OC axis, so that the two canonical H-bonds are deleted (Figure S7D) and the CH···O interaction is the only remaining H-bond in the dimer system.

**Table 1:** Interaction Energies (*E*_int_, kcal/mol) for Watson–Crick and Hoogsteen A–T Base Pairs for Echinomycin-Bound DNA (PDBID: 2ADW) and the NMR Structures for A6-DNA (PDBID: 5UZF) and m^1^A6-DNA (PDBID: 5UZI), along with AIM Electron Densities (au) for the H-Bond Path Critical Points

	*E*_int_ (kcal/mol)				ρ_BCP_ (au)		
	A–T pair	C–H to N^a^	change	90° rot^b^	A–NH···O–T	T–NH···N–A	A–CH···O–T
WC (2ADW)	13.06	10.51	–2.55	0.64	0.0308	0.0375	0.0047
Hoogsteen (2ADW)	13.79	10.46	–3.33	1.45	0.0242	0.0350	0.0045
WC (5UZF)	12.96	10.72	–2.24	–0.10	0.0281	0.0412	0.0036
Hoogsteen (5UZI)	13.54	9.71	–3.83	0.28	0.0246	0.0427	0.0042

a The change of C2/C8–H of adenine to N removes possible CH···O H-bonds.

b Rotation of T around φ(A–CH···OC–T) to 90° so as to destroy NH···O and NH···N HBs and retain only CH···O.

In addition to removing the canonical H-bonds, this rotation also takes the two bases out of their coplanar arrangement and so has an additional destabilizing impact on the system’s energy. Nonetheless, the interaction energy remains attractive by as much as 1.5 kcal/mol, which must be attributed to the remaining CH···O H-bond, and is in agreement with similar analyses^115,116^ performed on idealized A–T base pairs to determine the relative energy contributions of this bond. Altogether, the interaction energies from both the crystal and NMR (Table 1) coordinates are consistent with the existence of a weak CH···O H-bond. This is consistent with the frequency we observe in the duplexes (1720 cm^–1^) being greater than the value in water (1700 cm^–1^) and lower than that in *d*_6_-DMSO (∼1750 cm^–1^).

An atoms-in-molecules (AIM) analysis of the electron density at various H-bond critical points allows for an independent assessment of H-bond strengths, along with the H-bond lengths. As seen in Table 1, these densities are in the range of 0.025–0.043 au for the pair of canonical H-bonds, with NH···N slightly stronger than NH···O. But of the greatest importance here is that there is a bond path in all four systems linking the CH group with the O, consistent with its characterization as a H-bond. In keeping with the hypothesis that this bond is weaker than its canonical analogues, its critical point density is smaller but nonetheless falls within the range expected for a weak H-bond.

From these results, we conclude that there is a weak CH···O H-bond present in A–T WC and Hoogsteen base pairs. A few DFT studies on A–T WC base pairs have strongly disputed^52−54^ the existence of a CH2/8···O H-bond, but later studies have indicated that it is present^117,118^ although weak. Our experimental and theoretical results are in line with those of previous theoretical studies that support the existence of a third H-bond in A–T base pairs.

## 4 Discussion

Here, we describe a powerful method for isolating carbonyl vibrational frequencies from the highly congested IR spectra of DNA duplexes and DNA/drug complexes in aqueous solution. This in conjunction with plots relating the change in band position to the H-bonding strength of a solvent and allowing us to make qualitative statements about the H-bonding status of TC2=O in DNA duplexes in solution. A previous study^119^ incorporating modified adenines containing a N atom at the C2 position, which is incapable of H-bonding with TC2=O, reported a decrease in duplex melting temperature by 5 °C per modified base incorporated. The authors state that this may be due to interruption of the hydration spine of water present in duplexes or the repulsive interactions between the N and O atom lone pairs. Their observed decrease in the duplex melting temperature may also be due to interruption of a CH···O H-bond, the loss of which starts to destabilize the duplex. They noted that destabilization appeared to be independent of the sequence, which is consistent with our present results indicating that the position of the major TC2=O signal is unchanged with the sequence context. Base pair melting is an important parameter for cellular processes that require genomic DNA to be single-stranded such as replication, and thus this potential CH···O H-bond may supply a weak, sequence-independent energetic barrier for localized duplex melting.

Additionally, 2DIR studies also indicate that the TC2=O stretching motion may be a good reporter for minor groove interactions (such as with the spine of hydration) in general,^97^ and our results showing a red shift in this signal in our drug-bound duplexes support this conclusion. Every TC2=O stretching frequency in naked DNA duplexes that we have measured thus far has a frequency of 1720 cm^–1^ and so the “acceptor number” for TC2=O in a duplex environment (∼40) lies midway between those of DMSO (AN = 21) and water (AN = 54). While the adenine C2/H8 is not as strong as one of the most powerful H-bonders, liquid water, it is significantly stronger than DMSO. We therefore conclude that our observations thus far are consistent with a CH···O H-bond in A–T base pairs which is weak, but present, and could contribute to base-pair stability. Our DFT calculations support this conclusion and indicate that the energy of this bond (as compared to the unpaired A and T monomers) is ∼2 kcal/mol, supporting the conclusion that the bond is weak but certainly present and likely to contribute to the base pair’s stability.

It must be noted, however, that this is a qualitative interpretation as calculated T vibrational modes are well-known to change dramatically (particularly in the degree of their delocalization over multiple atoms)^88^ when going from a T monomer to the base-paired, duplex environment. Furthermore, at present, we cannot rule out H-bonding interactions with TC2=O from the spine of hydration^55−63^ observed in the minor groove in duplex crystal structures whose existence in solution is supported by a number of solution NMR^64,65^ and 2DIR^66−69^ experiments and molecular dynamics simulations.^70,71^ Thus, the position of the C2=O signal in our isotope editing IR experiments may arise from interaction with a weakly bound water molecule in the minor grove. However, the positions of the waters in the spine of hydration are thought to be sequence-dependent.^120−122^ If a water molecule from the spine of hydration were to impact the position of the IR signal, then we would have expected increases or decreases in wavenumber as a function of sequence as TC2=O experiences increases or decreases in H-bonding. We do not observe such changes, supporting the conclusion that there is a weak CH···O interaction between TC2=O and adenine.

## 5 Conclusions

In summary, we have demonstrated that ^13^C isotope editing at TC2=O can be used to isolate the stretching frequency of this carbonyl from the congested IR spectrum of DNA duplexes and DNA/drug complexes. Our solvatochromatic experiments combined with our isotope editing results are thus far consistent with a weak CH···O interaction with the TC2=O bond in both Watson–Crick and Hoogsteen A–T base pairs. Future work will employ labeling with isotopes at different positions to assign the major signal to the TC2=O signal in a DNA duplex and variable temperature studies to quantify the relationship between H-bond enthalpy and carbonyl stretches in DNA bases. D_2_O exchange studies will be conducted to probe the exposure of TC2=O to solvent waters. These may be combined with isotope editing experiments utilizing C2/8-D labeled adenine to observe mass shifts and determine the extent to which this TC2=O bond is H-bonded to water vs the adenine C2/8H.

## Supporting Information Available

Synthesis of ^13^C2-labeled thymine phosphoramidite; crystallographic information for the 9-methyladenine/1-methylthymine dimer; full IR spectrum of unlabeled TA-DNA and the fully ^15^N, ^13^C labeled and ^13^C2 labaled T isotopes; full IR spectra of free and bound AT-DNA; overlays of the isotope-edited spectra for all DNA duplexes examined in this work; observed density map for crystals of the N9-methyladenine and N1-methylthymine Hoogsteen dimers; and models and controls used in the ab initio calculations for Watson–Crick and Hoogsteen A–T base pairs (PDF)

## Notes

Atomic coordinates for the 9-methyladenine/1-methylthymine crystal have been deposited in the Cambridge Crystallographic Data Centre under deposition number 2053806.

## Abbreviations

T: thymine
A: adenine
C: cytosine
G: guanine
WC: Watson–Crick
