# Supplementary material for: Probing the Hydrogen-Bonding Environment of Individual Bases in DNA Duplexes with Isotope-Edited Infrared Spectroscopy
Source: J Phys Chem B. 2021 Jul 8;125(28):7613–27. doi: 10.1021/acs.jpcb.1c01351 (PMC8311644; doi:10.1021/acs.jpcb.1c01351)
Supplement: Supplementary file 1 — jp1c01351_si_001.pdf [file jp1c01351_si_001.pdf]

Supporting Information for:

Probing the hydrogen bonding environment of individual bases in DNA duplexes with isotope edited infrared spectroscopy

*Robert J. Fick[1], Amy Y. Liu[2], Felix Nussbaumer[3], Christoph Kreutz[3], Atul Rangadurai[2], Yu Xu[4], Roger D. Sommer[5], Honglue Shi[4], Steve Scheiner[6], Allison L. Stelling[1,2]**

[1]Department of Chemistry and Biochemistry, The University of Texas at Dallas, Richardson TX, 75080 USA

[2]Department of Biochemistry, Duke University Medical Center, Durham NC, 27710 USA

[3]Institute of Organic Chemistry and Center for Molecular Biosciences Innsbruck (CMBI), University of Innsbruck, Innsbruck, Austria

[4]Department of Chemistry, Duke University, Durham, NC, 27710 USA

[5]Molecular Education, Technology, and Research Innovation Center, North Carolina State University, Raleigh NC, 27695 USA

[6]Department of Chemistry and Biochemistry, Utah State University, Logan Utah, 84322 USA

* To whom correspondence should be addressed: Tel: (972) 883-6718, Email: stelling@utdallas.edu

SUPPORTING METHODS

Synthesis of ^{13}C2 labeled thymine phosphoramidite

*(1-^{13}C)-N-Carbamoyl-2-cyanacetamid (**1**):* 2-Cyanoacetic acid (3.09 g, 30.8 mmol), ^{13}C-urea (2.04 g, 33.9 mmol) and acetic anhydride (4.72 g, 46.2 mmol) were heated with stirring to 90°C for 1h. After 15min a white precipitate was formed. After cooling to room temperature, 200 mL of diethyl ether was added and the formation of a suspension was completed by treating the reaction mixture in an ultrasonic bath. Solid **2** was filtered off and dried in high vacuum. Yield: 3.19 g (81 %) as a pale yellow solid. ^{1}H-NMR (300 MHz, DMSO-d_6, 25°C): δ 3.93 (q, 1H, *H*C); 1.41 (d, 3H, C*H*$_3$) ppm. ^{13}C-NMR (75 MHz, DMSO-d_6, 25°C): δ 153.06 (^{13}COOH); 118.51 (CN); 32.85 (CH); 15.37 (CH_3) ppm.

*(2-^{13}C)-Thymine (**2**):* Pd/$BaSO_4$ (5%, 800 mg) was suspended in 10 mL 50% aqueous acetic acid in a 500 mL three-necked round bottom flask. The palladium catalyst was treated several times with hydrogen by evacuating the flask followed by spilling with hydrogen. Simultaneously, compound **1** (1.76g, 12.4 mmol) was dissolved in 40 mL of boiling 50% acetic acid and then added to the reduced palladium catalyst. The reaction mixture was stirred at room temperature under a hydrogen atmosphere for 16h. Before being filtered through celite, the mixture was heated to 70°C for 1h. The solvent was evaporated until a white precipitate was formed. Precipitation of 3 was completed by storing the suspension at 4°C overnight. Compound **2** was filtered off and dried in high vacuum overnight. Yield: 1.06 g (67 %) as a pale yellow solid. ^{1}H-NMR (300 MHz, DMSO-

d_6, 25°C): δ 10.73(bs, 2H, 2 x N*H*); 7.23(d, 1H, *H*C6); 1.72 (d, 3H, CH_3) ppm. ^{13}C-NMR (75 MHz, DMSO-d_6, 25°C): δ 164.93 (C4); 151.50 (^{13}C2); 137.74 (C6); 107.72 (C5); 11.79 (CH_3) ppm.

3´,5´-O-Bis toloyl-(2-^{13}C)-2´-deoxythymidine (3): Compound **2** (2.11 g, 16.6 mmol) together with hexamethyldisilazane (50 mL) and trimethylsilylchloride (5.0 mL) was refluxed under an argon atmosphere at 120 °C overnight. After cooling to room temperature, the mixture was evaporated to an oily residue, which was co-evaporated with a small amount of dry chloroform and then dried in high vacuum for 30 min. The oil was dissolved in 60 mL of dry chloroform, Hoffer´s α;-chlorosugar (5.81 g, 14.94 mmol, 1-chloro-2-deoxy-3,5-di-*O*-toluoyl-α;-*D*-ribofuranose) was added at once and the mixture stirred for 4h at 40°C. After cooling to room temperature, the mixture was diluted with methylene chloride and washed twice with saturated sodium bicarbonate solution. The organic phase was dried over sodium sulfate and evaporated to dryness. The solid residue was re-crystallized from boiling ethanol and dried in high vacuum to give **3**. Yield: 4.44 g (56%) as a white, crystalline solid. TLC: (CH_2Cl_2/MeOH = 9/1) R_f = 0.7 ^{1}H-NMR (300 MHz, $CDCl_3$, 25°C): δ 8.97 (s, 1H, N*H*); 7.95, 7.29 (m, 8H, C*H*ar); 6.47 (m, 1H, C1'*H*); 5.65 (d, 1H; C3'*H*); 4.81, 4.67 (m, 2H, C5'*H*); 4.54(s, 1H, C4'*H*); 2.72, 2.33 (m, 2H, C2'H_2); 2.45(s, 6H, 2 x ar-CH_3); 1.65(s, 3H, C5-CH_3) ppm. ^{13}C-NMR (75 MHz, $CDCl_3$, 25°C): δ 166.22 (C q); 166.16 (C q); 163.59 (C4 q); 150.42 (^{13}C2); 144.69 (C q); 134.53 (C6); 129.96 (CH ar); 129.64 (CH ar); 129.59 (CH ar); 129.41 (CH ar); 126.68 (C q); 126.40 (C q); 111.78 (C5H); 85.06 (C1'); 82.92 (C4'); 75.02 (C3'); 64.30 (C5'); 38.18 (C2'); 21.84 (Car-CH_3); 21.80 (Car-CH_3; 12.23 (C5-CH_3) ppm.

(2-^{13}C)-2´-Deoxythymidine (4): Compound **3** (4.44 g, 9.29 mmol) was treated with 100 mL methylamine solution (33 wt % in absolute ethanol) and stirred at room temperature overnight. The reaction mixture was evaporated to an oily residue which was dissolved in a minimum (about

5 mL) of hot methanol. The methanolic solution was added with a pipette to 250 mL of a mixture of methylene chloride/diethyl ether/n-hexane (1/1/1) with stirring. A white precipitate was formed and precipitation was completed by storing the mixture 3h at 4°C. The suspension was filtered with suction, washed with methylene chloride and solid compound **5** was dried in high vacuum. Yield: 1.98 g (88 %) of a white solid. TLC: (CH_2Cl_2/MeOH = 9/1) R_f = 0.2 ^{1}H-NMR (300 MHz, DMSO-d_6, 25°C): δ 11.22 (bs, 1H, N*H*); 7.69 (s, 1H, C6*H*); 6.16 (m, 1H, C1'*H*); 5.21 (d, 1H, C3'O*H*); 4.99 (t, 1H, C5'O*H*); 4.23 (m, 1H, C3'*H*); 3.76 (dt, 1H, C4'*H*); 3.56 (m, 2H, C5'H_2); 2.06 (m, 2H, C2'H_2); 1.77 (d, 3H, CH_3) ppm. ^{13}C-NMR (75 MHz, $CDCl_3$, 25°C): δ 163.71 (C q); 150.44 (^{13}C2); 136.08 (C6); 109.32 (C5); 87.24 (C4'); 83.74 (C1'); 70.42 (C3'); 61.33 (C5'); 39.40 (C2'); 12.22 (CH_3) ppm.

*5'-O-(4,4-Dimethoxytrityl)(2-^{13}C)-2′-deoxythymidine (**5**):* Compound **5** (1.95 g, 8.05 mmol) together with one spatula (catalytic amount) of 4-(dimethylamino)pyridine was co-evaporated twice with anhydrous pyridine and then dissolved in 25 mL of anhydrous pyridine. Then 4,4'-dimethoxytrityl chloride (3.27 g, 9.66 mmol) was added in three portions within one hour and the mixture was stirred 3 h at room temperature or until TLC showed complete conversion. The mixture was quenched with 2 mL of methanol, evaporated to an oily residue and two times co-evaporated with toluene. The orange foam was dried in high vacuum for 30 min and dissolved in methylene chloride. The organic phase was washed two times with 5 % citric acid, once with saturated sodium bicarbonate solution, dried over sodium sulfate and evaporate to dryness. The crude product was applied onto a silica gel column with methylene chloride and eluted with a gradient from 0% to 5% methanol in methylene chloride to give pure **5**. Yield: 3.11 g (71 %) as a yellowish foam. TLC: (CH_2Cl_2/MeOH = 9/1) R_f = 0.4 ^{1}H-NMR (300 MHz, $CDCl_3$, 25°C): δ 9.27 (bs, 1H, N*H*); 7.61 (d, 1H,C6*H*) 7.45 – 7.29 (m, 9H, C*H*ar); 6.88 – 6.85 (m, 4H,

C*H*ar); 6.45 (t, 1H, C1'*H*); 4.60 (m, 1H, C3'*H*); 4.09 (m, 1H, C4'*H*); 3.80 (s, 6H, 2 x O-CH_3); 3.47 (m, 2H, C5'H_2); 2.97 (d, 1H, C3'O*H*); 2.38 (m, 2H, C2'H_2); 1.50 (d, 3H, $^3J_{CH}$ = 6.3 Hz, CH_3) ppm. ^{13}C-NMR (75 MHz, $CDCl_3$, 25°C): δ 163.93 (C q); 158.86 (C q); 150.70 (^{13}C2); 135.77 (C6); 135.55 (C q); 135.50 (C q); 130.21 (CH ar); 128.26 (CH ar); 128.12 (CH ar); 127.27 (C q); 113.42 (CH ar); 111.42 (C5); 86.34 (C4'); 84.89 (C1'); 72.64 (C3'); 63.73 (C5'); 55.38 (2 x OCH_3); 41.08 (C2'); 11.95 (-CH_3) ppm.

*5'-O-(4,4'-dimethoxytrityl)-(2-^{13}C)-2'-deoxythymidine-3'-O-[O-(2-cyanoethyl)-N,N'-diisopropylphosphoramidite] (**6**):* Compound **5** (3.05 g, 5.61 mmol) together with *N*,*N*-diisopropylethylamine (56.1 mmol, 7.24 g) was dissolved in 50 mL of dry methylene chloride and sealed with a septum under an argon atmosphere. Then, 2-cyanoethyl-*N*,*N*-diisopropylchlorophosphoramidite (CEP-Cl, 6.73 mmol, 1.59 g) was added with a syringe and the mixture was stirred at room temperature for 3h or until TLC showed complete conversion. The reaction was quenched by adding 2 mL of dry methanol and stirring was continued for 10 minutes. The mixture was diluted with methylene chloride, washed with saturated sodium bicarbonate solution, the organic phase was dried over sodium sulfate, evaporated to dryness and dried in high vacuum for 30 minutes. The crude product was applied onto a silica gel column and eluted with a mixture of hexane/ethylacetate (+ 3% triethylamine) to give pure **6**. Yield: 3.09 g (74 %) of a colorless foam. TLC: (ethylacetate / n-hexane = 1/1 + 3 % NEt_3) R_f = 0.7 ^{1}H-NMR (300 MHz, DMSO-d_6, 25°C): δ 8.72 (bs, 1H, N*H*); 7.60 (dd, 1H, C6*H*); 7.4 – 7.2 (m, C*H* ar); 6.85 – 8.81 (m, C*H* ar); 6.40 (m, 1H, C1'*H*); 4.64 (m, 1H, C3'*H*); 4.16(m, 1H, C4'*H*); 3.66 (m, 2H, -CH_2OP); 3.46 (m, 2H, C5'H_2); 2.62 – 2.42 (m, 2H, -CH_2CN); 2.44 (m, 2H, C2'H_2); 1.45 (d, 3H, $^3J_{CH}$ = 6.3 Hz, CH_3); 1.17 (d, 14H, iPr-C*H*, iPr-CH_3) ppm. ^{13}C-NMR (75 MHz, $CDCl_3$, 25°C): δ 150.49, 150.42 (^{13}C2) ppm. ^{31}P-NMR (121 MHz, $CDCl_3$, 25°C): δ 149.62; 149.19 ppm.

SUPPORTING TABLES AND FIGURES

Supporting Table 1: Crystallographic information for the 9-methyladenine/1methylthymine dimer.

Formula	$C_{12}H_{15}N_7O_2$
M_r	289.31
Crystal system, sp. Group	Monoclinic, $P2_1/m$
Temp. (K)	100(2)
a, *b*, *c* (Å)	8.312(3), 6.544(2), 12.857(4)
α, β, γ (°)	90, 106.777(10), 90
V (Å^3)	669.6(3)
Z	2
μ (mm^{-1})	0.104
Crystal size (mm)	0.096× 0.212 × 0.572
Refl. measured, indep.,	12262, 2220, 1680
Completeness	0.998
R_{int}	0.0230
(sin θ/λ)$_{max}$ (Å^{-1})	0.751
R_1^a, wR_2^b, S^c	0.0426, 0.1197, 1.012
$\Delta\rho_{max}$, $\Delta\rho_{min}$ (e/Å^{-3})	0.358, −0.195

^{a} $R_1 = \Sigma(||F_o| - |F_c||) / \Sigma|F_o|$ for $[F^2 > 2\sigma(F^2)]$, ^{b} $wR_2 = [\Sigma (w(F_o^2 – F_c^2)^2) / \Sigma (w(F_o^2)^2]^{½}$;
$w=1/[\sigma^2(F_o^2)+(aP)^2+bP]$,
^{c}GOF= $\{\Sigma[w(F_o^2 - F_c^2)^2] / (\#reflns - \#parms)\}^{½}$

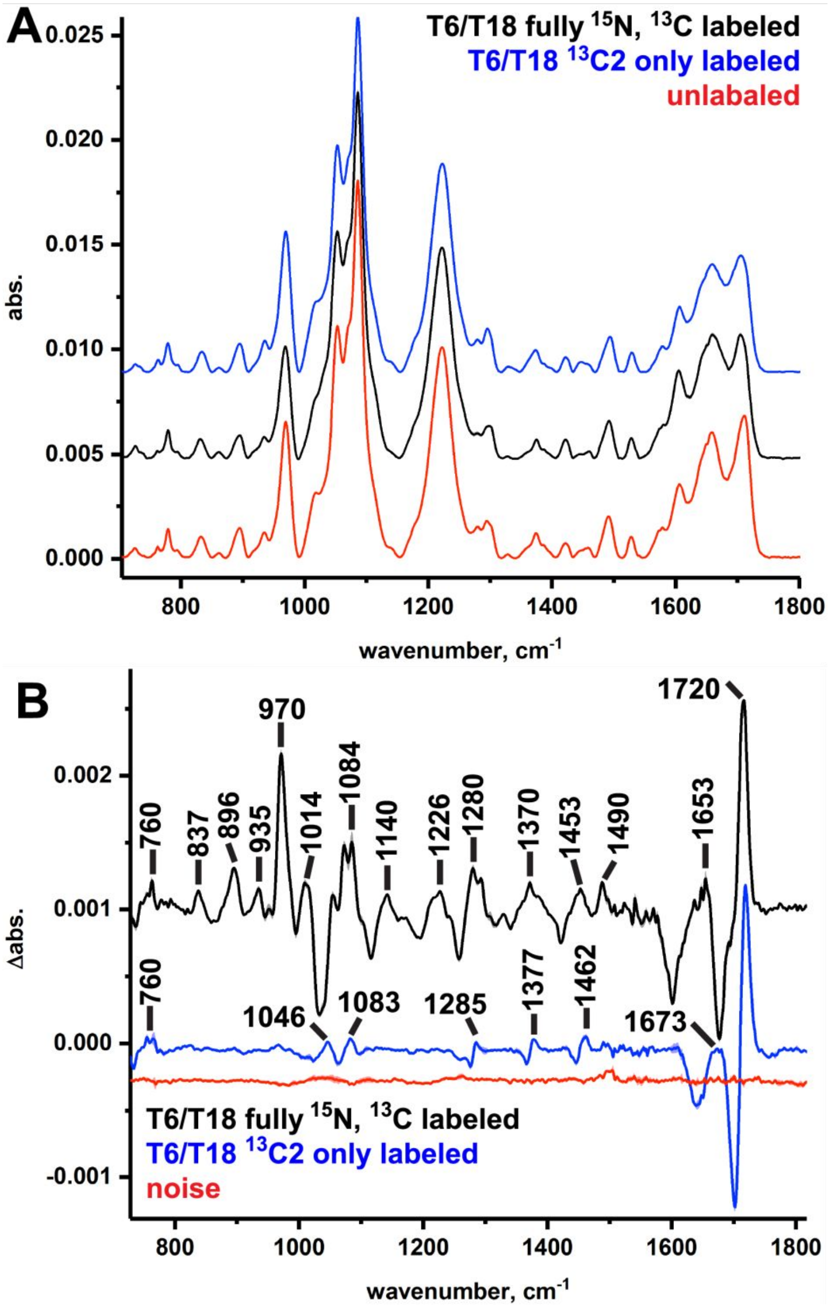

Supporting Figure 1: (A) Full IR spectrum of unlabeled TA-DNA (red) and two T isotopes incorporated into the 6 and 18 positions: the fully labeled ^{13}C, ^{15}N T base (black) and the $^{13}C2$ T base (blue). (B) Difference spectra taken between the unlabeled TA-DNA and the C2 labeled isotope (blue) the fully labeled T isotope (black). The noise line (red) is the average of multiple replicates subtracted from each other. Mean is the bold and standard deviation in shade.

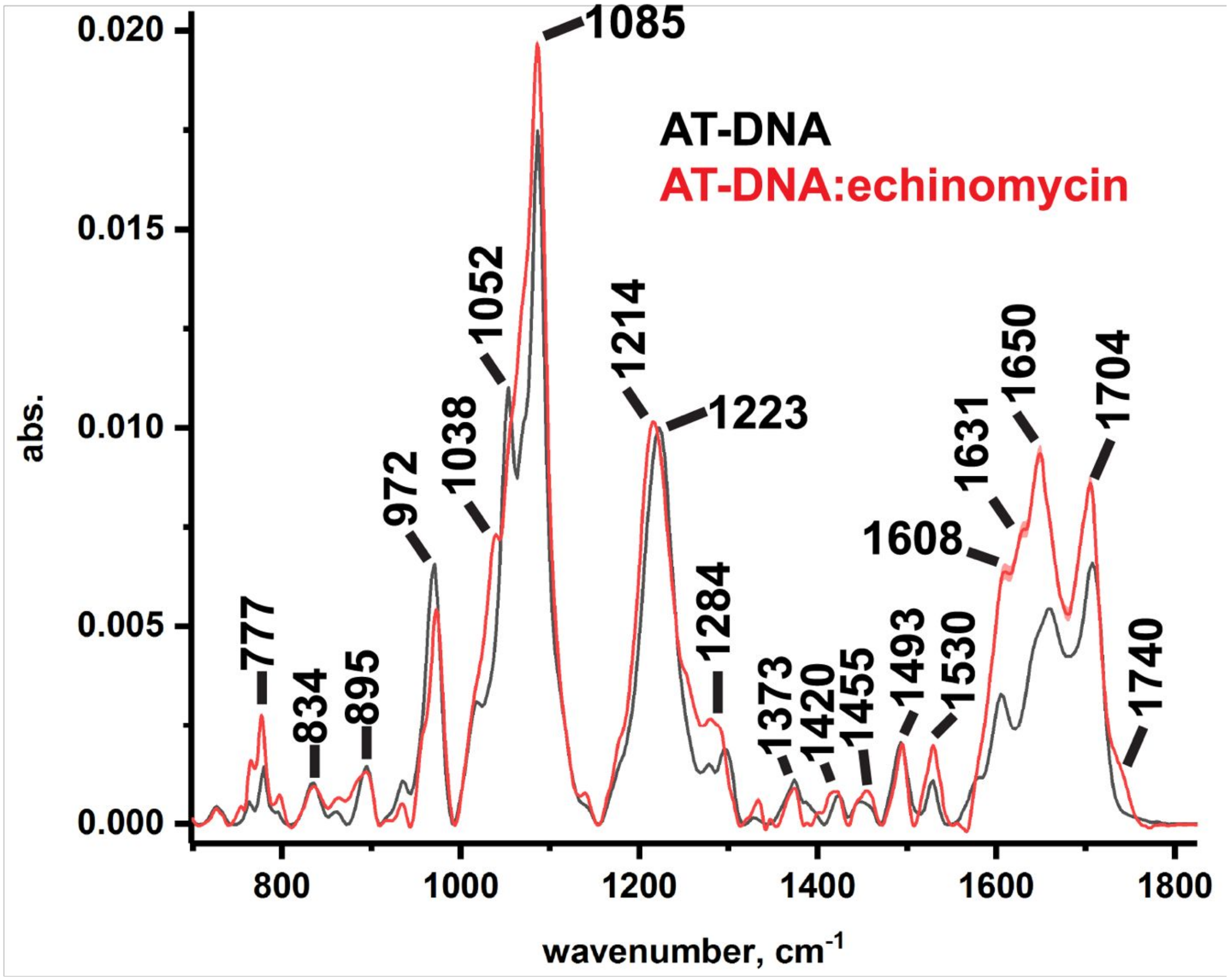

Supporting Figure 2: Full IR spectra for AT-DNA when free (black) and when bound to echinomycin (red) showing the characteristic changes upon drug binding. Mean in of three replicates in bold and standard deviation in shade.

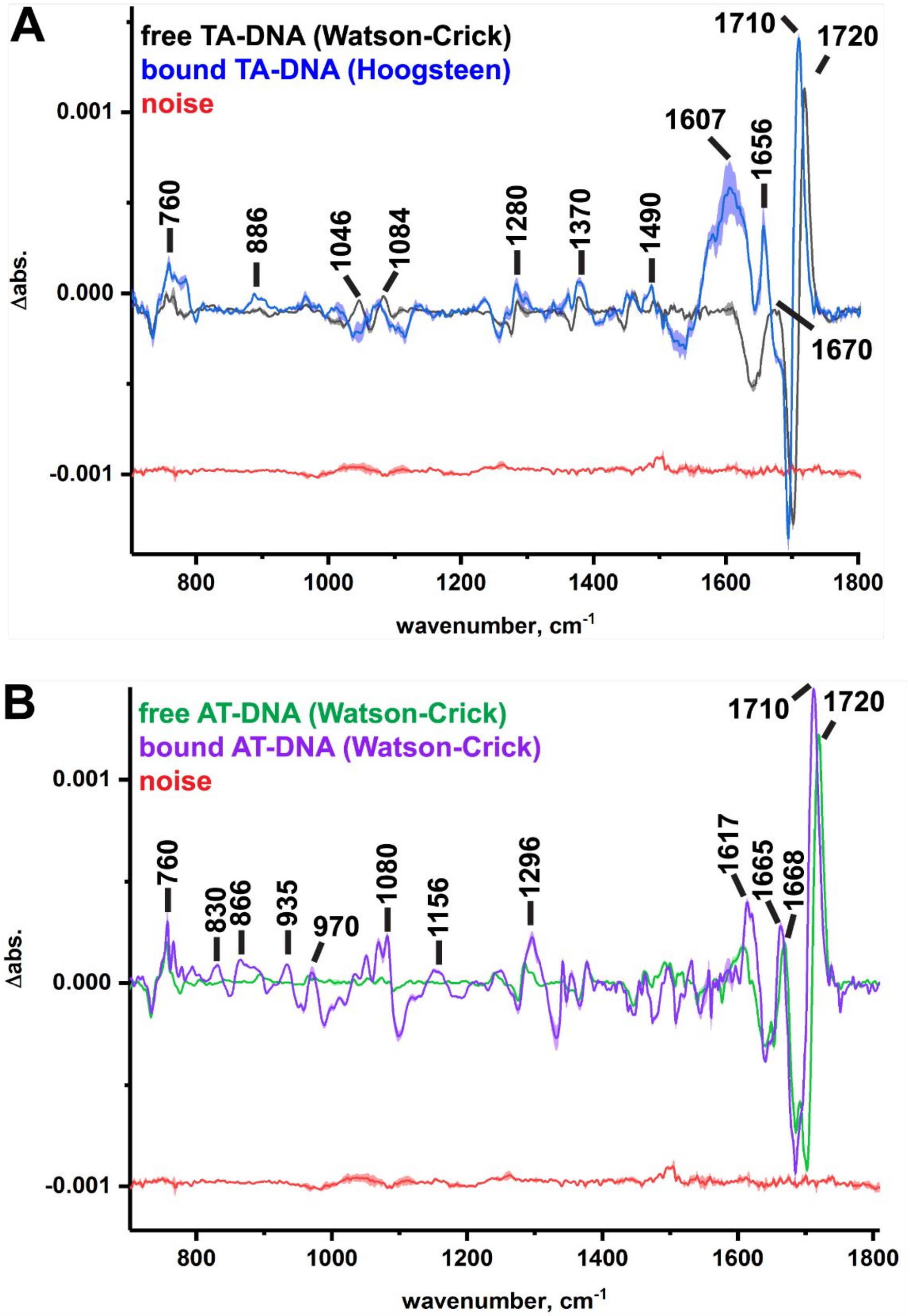

Supporting Figure 3: Overlays of the isotope edited spectra of free and echinomycin bound (A) TA-DNA and (B) AT-DNA and a representative noise line (replicate minus replicate for the free TA-DNA).

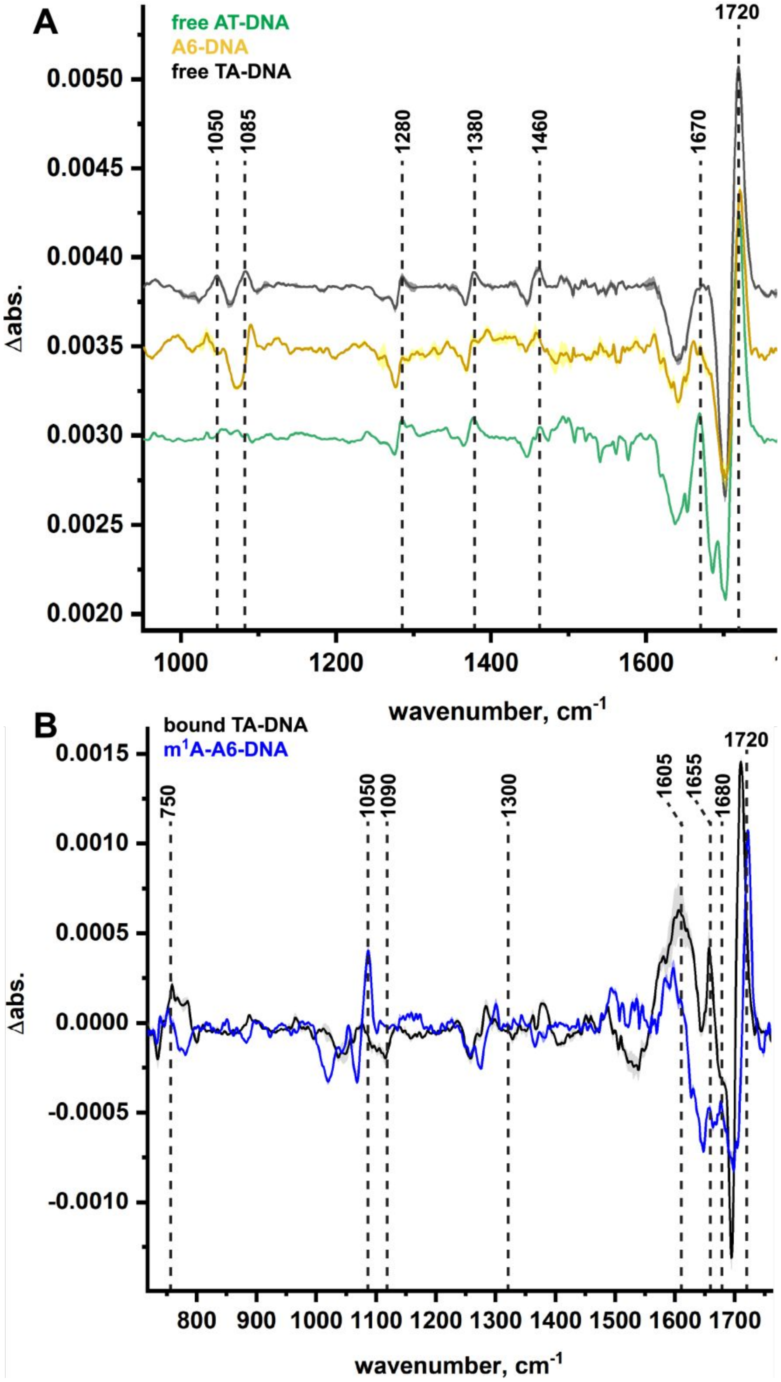

Supporting Figure 4: Overlays of isotope edited spectra for (A) Watson-Crick A-T base pairs and (B) Hoogsteen base pairs.

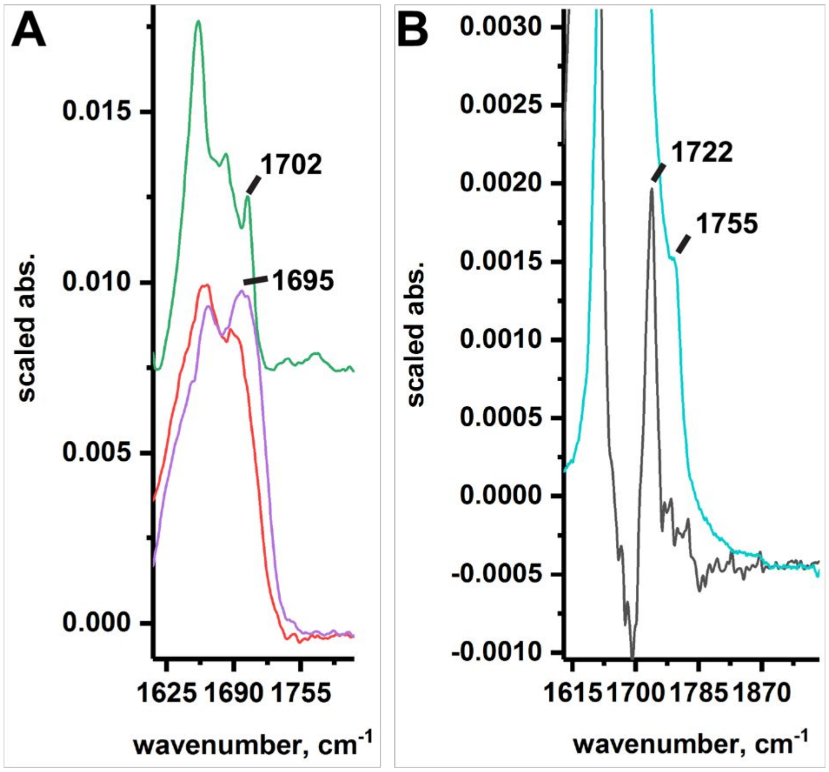

Supporting Figure 5: Carbonyl region for (A) single stranded 12-mer of poly-T (red) and N1-methylthymine (purple) in water and the A-T Hoogsteen dimer crystal (green) and (B) N1-methylthymine in d6-DMSO (cyan) and DMF (black).

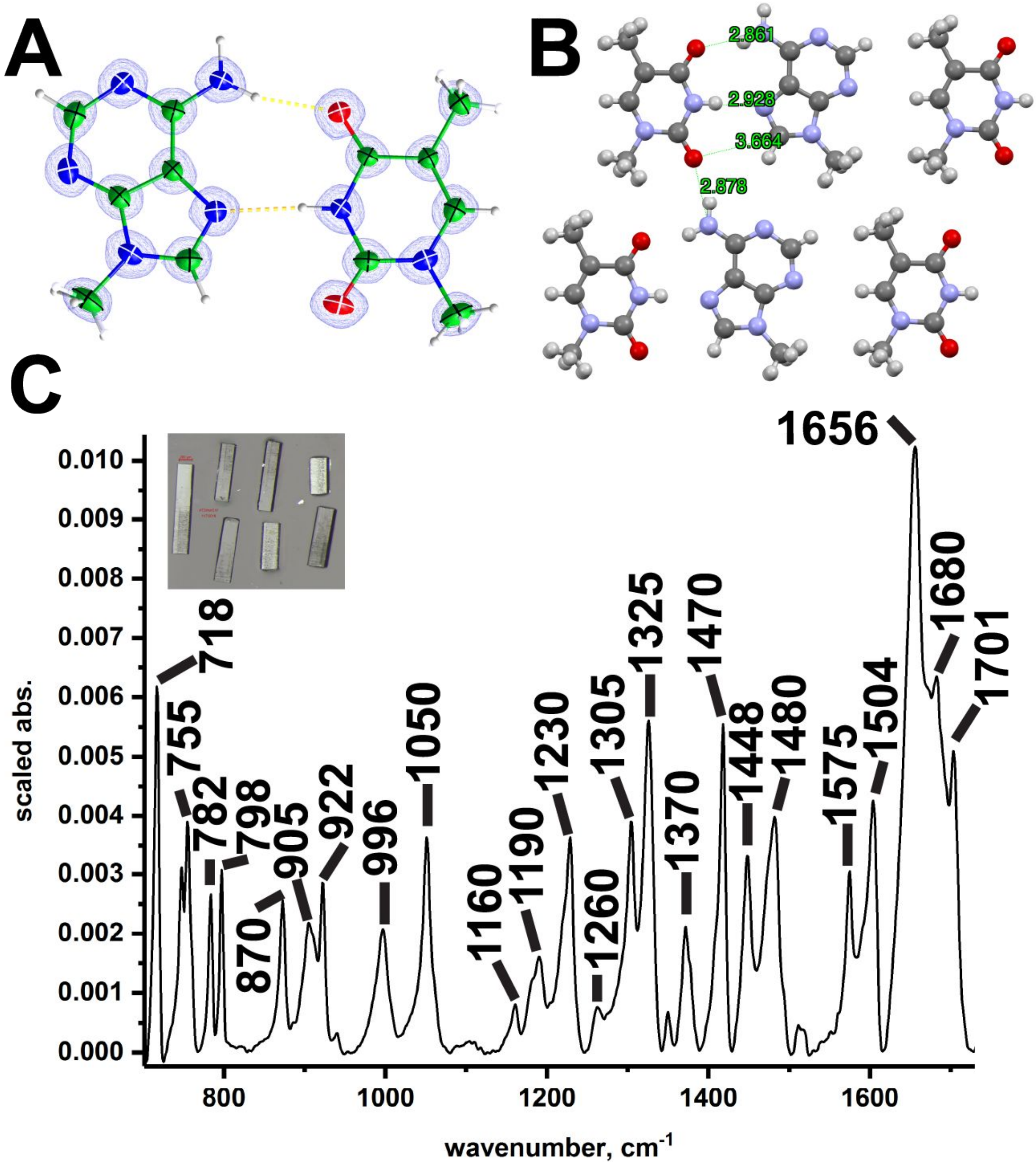

Supporting Figure 6: (A) The observed density map for crystals of the N9-methyladenine and N1-methylthymine Hoogsteen dimer. (B) Crystal packing of the dimer showing interactions of the C2=O with atoms in neighboring unit cells. (C) IR spectrum collected from the Hoogsteen dimer crystals (shown in inset). Distances are in angstroms.

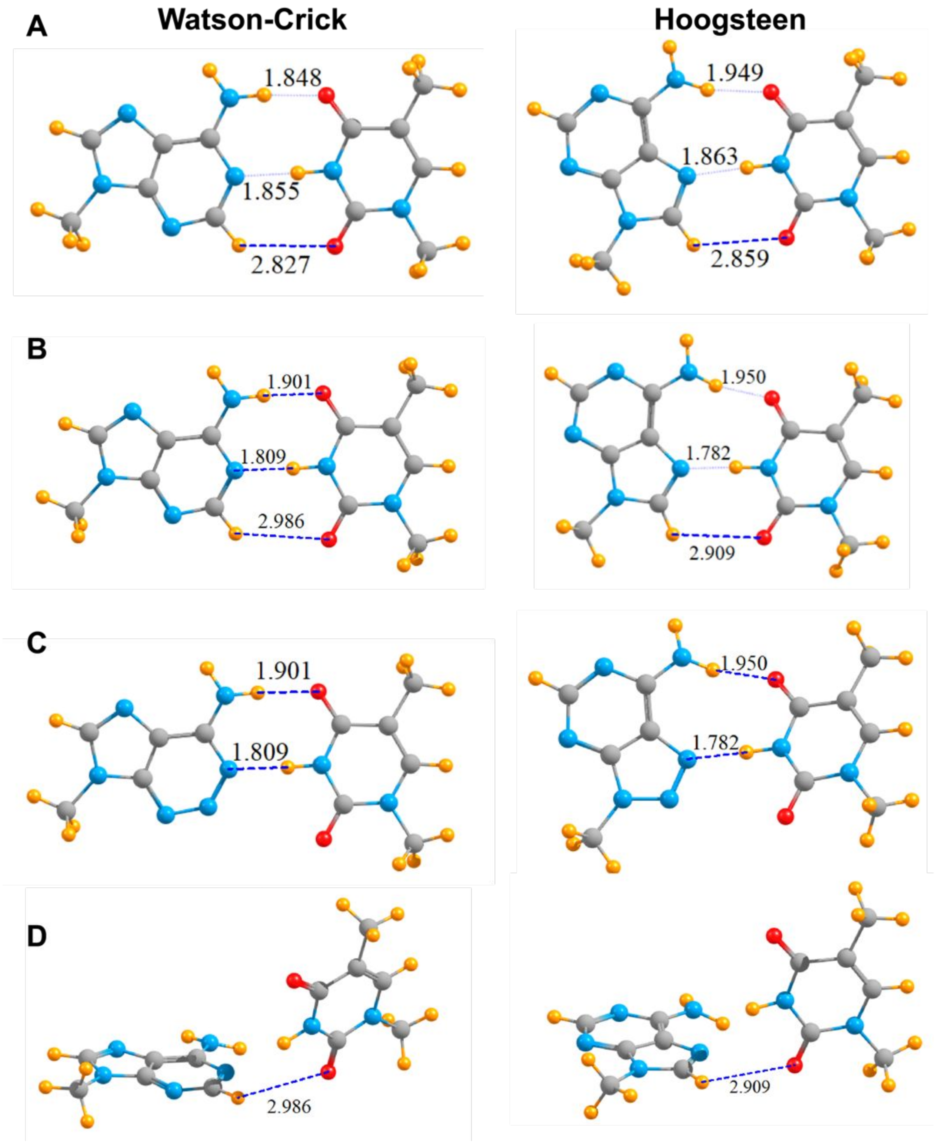

Supporting Figure 7: Models and controls used in the *ab initio* calculations for Watson-Crick and Hoogsteen A-T base pairs with distances (in angstroms) between the H-bonding atoms shown. (A) Optimized Watson-Crick and Hoogsteen base pairs taken from the crystal structure (PDBID: 2ADW) coordinates. (B) Optimized Watson-Crick and Hoogsteen base pairs taken from the NMR coordinates for A6-DNA (PDBID: 5UZF) and m^1A-A6-DNA (PDBID: 5UZI). The methyl group has been removed in the m^1A-A6-DNA model. (C) Optimized coordinates from A6-DNA in which an N atom replaces the C2 (in the Watson-Crick dimer) or C8 (in the Hoogsteen dimer) atom, abolishing the CH···O bond. (D) Optimized coordinates from the m^1A-A6-DNA duplex in which the T base is rotated by 90° to abolish the two canonical H-bonds in the A-T dimer and leave only the CH···O bond.
